# Spermatogenic cell-specific type 1 hexokinase (HK1S) is essential for capacitation-associated increase in tyrosine phosphorylation and male fertility in mice

**DOI:** 10.1371/journal.pgen.1011357

**Published:** 2024-07-29

**Authors:** Yingchao Tian, Xiu Chen, Jie Pu, Yuxin Liang, Weixi Li, Xiaotong Xu, Xinshui Tan, Shuntai Yu, Tianyu Shao, Yan Ma, Bingwei Wang, Yongjie Chen, Yushan Li

**Affiliations:** 1 The School of Public Health, Xinxiang Medical University, Xinxiang, Henan, China; 2 Department of Pharmacy, Heze University, Heze, Shandong, China; 3 Department of Pharmacology, Nanjing University of Chinese Medicine, Nanjing, China; 4 National Institute of Biological Sciences, Beijing, Beijing, China; 5 Central Laboratory, Beijing Obstetrics and Gynecology Hospital, Capital Medical University, Beijing Maternal and Child Health Care Hospital, Beijing, China; HudsonAlpha Institute for Biotechnology, UNITED STATES OF AMERICA

## Abstract

Hexokinase (HK) catalyzes the first irreversible rate-limiting step in glycolysis that converts glucose to glucose-6-phosphate. HK1 is ubiquitously expressed in the brain, erythrocytes, and other tissues where glycolysis serves as the major source of ATP production. Spermatogenic cell-specific type 1 hexokinase (HK1S) is expressed in sperm but its physiological role in male mice is still unknown. In this study, we generate *Hk1s* knockout mice using the CRISPR/Cas9 system to study the gene function *in vivo*. *Hk1s* mRNA is exclusively expressed in testes starting from postnatal day 18 and continuing to adulthood. HK1S protein is specifically localized in the outer surface of the sperm fibrous sheath (FS). Depletion of *Hk1s* leads to infertility in male mice and reduces sperm glycolytic pathway activity, yet they have normal motile parameters and ATP levels. In addition, by using *in vitro* fertilization (IVF), *Hk1s* deficient sperms are unable to fertilize cumulus-intact or cumulus-free oocytes, but can normally fertilize zona pellucida-free oocytes. Moreover, *Hk1s* deficiency impairs sperm migration into the oviduct, reduces acrosome reaction, and prevents capacitation-associated increases in tyrosine phosphorylation, which are probable causes of infertility. Taken together, our results reveal that HK1S plays a critical role in sperm function and male fertility in mice.

## Author summary

About 8%-12% of couples worldwide are infertile, and male infertility is associated with about half of these cases. Common defects of male infertility include low sperm count, reduced sperm motility, abnormal sperm morphology, and unexplained male infertility with normal sperm parameters but infertility. The cause of over 50% of male infertility cases remains unidentified. Research on male reproduction has helped to unravel male infertility. In this study, we used a knockout mouse model to study the function of HK1S in the male mouse reproductive system. We showed that depletion of *Hk1s* leads to infertility in male mice but with normal sperm morphology and motility. These phenotypes are similar to patients with unexplained male infertility. Furthermore, HK1S is essential for sperm glycolysis. *Hk1s* deficiency in mice results in decreased glycolysis activity and leads to impaired sperm migration into the oviduct, reduced acrosome reaction, and defective capacitation-associated increase in tyrosine phosphorylation. These results suggested that HK1S is necessary for male fertility in mice. Our study suggests a new avenue for the development of new therapeutic strategies for male infertility.

## Introduction

Mammalian sperm leaving the male reproductive tract after ejaculation are functionally immature, exhibit progressive motility, and cannot successfully fertilize oocytes. To acquire fertilizing ability, sperm must undergo several biochemical and functional changes in the female reproductive tract, collectively known as capacitation [1,2]. During capacitation, sperm switch from progressive to hyperactivated motility, and undergo the acrosome reaction [2,3]. These processes allow sperm to penetrate the cumulus cell layer and zona pellucida (ZP) of the oocyte to complete fertilization [3], whereas defects in these events can finally lead to subfertility or infertility.

Hexokinase (HK) is an enzyme that catalyzes the first committed step of glycolysis, which utilizes ATP to phosphorylate glucose and produce glucose-6-phosphate (G6P). G6P can enter the glycolytic pathway for energy production, the pentose phosphate pathway (PPP) for anabolic intermediates, or be converted to glucose-1-phosphate for glycogen synthesis [4,5]. In mammals, five HK isozymes (HK1, HK2, HK3, glucokinase [GCK], and HK domain-containing 1 [HKDC1]) have been identified, each with distinct tissue expression, subcellular localization and enzyme kinetics [4,6]. HK1 is a ubiquitous enzyme that is present in most cell types, especially at the highest levels in brain and erythrocytes [7,8]. It contains an N-terminal 20-amino acid hydrophobic sequence termed porin-binding domain (PBD) that binds HK1 to voltage-dependent anion channels (VDACs) in the outer mitochondrial membrane [4,9]. However, three variant transcripts of *Hk1* (*Hk1_v1*, *Hk1_v2*, and *Hk1_v3*) are expressed specifically in spermatogenic cells, and named spermatogenic cell-specific type 1 hexokinase (HK1S). HK1S encodes a different N-terminal 24-amino acid sequence, called spermatogenic cell-specific region (SSR), which replaces the PBD of the HK1 isoform present in somatic cells [8,10,11]. HK1S is tethered in the principal piece region by a spermatogenic cell-specific muscle-type phosphofructokinase variant isozyme (PFKMS), which in turn is tightly bound to glutathione S-transferase mu class 5 (GSTM5) in the fibrous sheath (FS) [12].

Glycolysis and oxidative phosphorylation (OXPHOS) are the two major metabolic pathways producing ATP which is the primary source of energy for sperm, with glycolysis occurring in the FS of the principal piece, and OXPHOS occurring in mitochondria that are exclusively localized in the midpiece of flagellum [13,14]. In some mammals, such as humans and mice, ATP is mainly produced by glycolysis [15,16], and most of the glycolytic enzymes have unique structures and functions. Some are products of alternative transcript splice variants of genes expressed in other cells, such as HK1S [8,10,17], PFKMS [12], and aldolase (ALDOA_V2) [18]. Others are products of spermatogenic cell-specific genes, including glyceraldehyde 3-phosphate dehydrogenase, spermatogenic (GAPDHS) [19,20], lactate dehydrogenase-C4 (LDHC) [21], phosphoglycerate kinase 2 (PGK2) [22], two aldolase A (Aldoa)-related retrogenes (ALDOART1 and ALDOART2) [18]. Several glycolytic enzymes are tightly bound to the FS of mouse sperm [23]. Previous *in vitro* studies of mouse and human sperm indicate that glycolysis produces large amounts of ATP [16,24,25] and is required for multiple steps of fertilization, including capacitation-dependent tyrosine phosphorylation [26,27], sperm motility [16,28], and penetration of the ZP [29]. *In vivo* studies targeting disruption of glycolytic isozyme *Gapdhs*, *Ldhc* or *Pgk2* respectively result in reduced levels of ATP in sperm, disruption of sperm motility, abnormal tyrosine phosphorylation and male infertility [30–32], confirming that glycolytic enzymes are essential for sperm function and fertilization in mice. However, the role of HK1S in male fertility in mammals is still unknown.

Here, seeking to investigate the role of the mouse *Hk1s* gene in male fertility, we generate an *Hk1s* knockout mouse line using the CRISPR/Cas9 system. The ablation of *Hk1s* in mice leads to male infertility and reduces glycolytic pathway activity. In addition, IVF using *Hk1s* deficient sperms cannot fertilize cumulus-intact or cumulus-free oocytes but is able to successfully fertilize zona pellucida-free oocytes. Furthermore, *Hk1s* deficiency impairs sperm migration into the oviduct, acrosome reaction, and capacitation-associated increases in tyrosine phosphorylation. Collectively, our findings demonstrate that HK1S is indispensable for male fertility.

## Results

### Expression of *Hk1s* in adult mice and generation of *Hk1s* knockout mice

**Fig 1:**
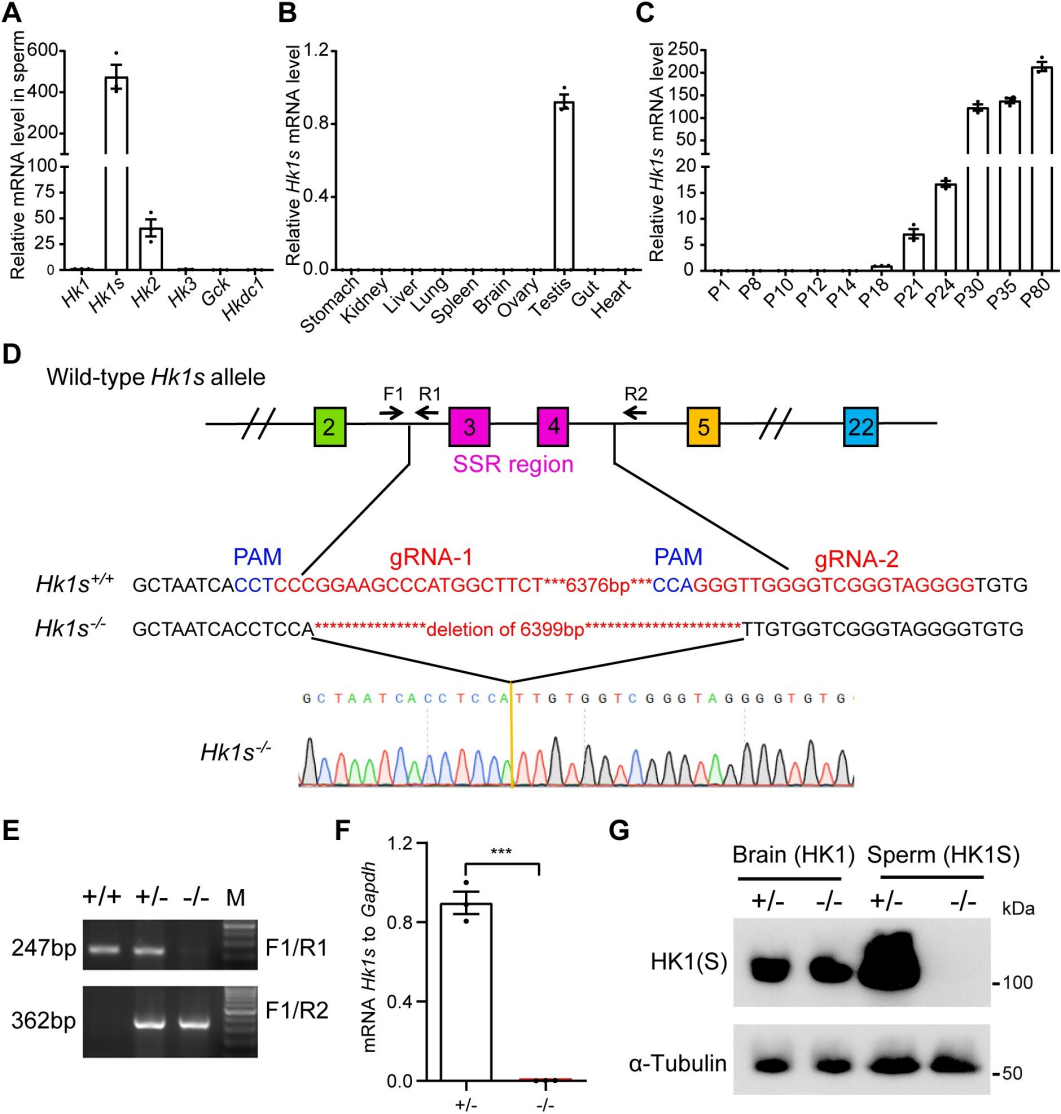
Expression pattern of *Hk1s* in mice and generation of *Hk1s*^−/−^ mice. (A) qRT-PCR analysis of transcript levels of members of the HK gene family in mouse sperm. Mice number (n = 3). (B) qRT-PCR analysis of *Hk1s* mRNA levels in various organs of adult mice. Mice number (n = 3). (C) qRT-PCR analysis of *Hk1s* mRNA levels in mouse testes of indicated ages. Mice number (n = 3). (D) Schematic diagram showing the gene structure of *Hk1s* and the CRISPR/Cas9 system used to generate the knockout allele. The upper panel shows the region of the *Hk1s* locus that was targeted. Two gRNAs were used to achieve deletion of the SSR genomic fragment (6399bp). The frameshift-mutated sequences of the knockout allele and Sanger sequencing are shown in the middle and lower panels. The locations of the gRNAs and primers (F1, R1, and R2) are indicated. (E) Genotyping of *Hk1s*^−*/*−^ mice by primers indicated in (D); the sizes of PCR products are shown on the left. (F) qRT-PCR analysis of *Hk1s* mRNA levels in *Hk1s*^+*/*−^ and *Hk1s*^−*/*−^ sperm. Mice number (n = 3) per genotype. Error bars: SEM. Statistics, Student’s *t*-Test. ***, *P* < 0.001. (G) Western blot analysis of HK1 and HK1S protein levels in *Hk1s*^+*/*−^ and *Hk1s*^−*/*−^ brain and sperm. α-Tubulin was used as a loading control.

We initially examined the transcript levels of the HK gene family members in sperm from the adult mouse cauda epididymis using quantitative real-time PCR (qRT-PCR). We found that *Hk1s* was highly expressed and *Hk2* was moderately expressed in sperm, while *Hk1*, *Hk3*, *Gck* and *Hkdc1* were undetectable in sperm (Fig 1A). We then analyzed *Hk1s* mRNA transcript levels in various adult mouse tissues and found that *Hk1s* was restricted to the testis (Fig 1B). To determine the expression pattern of *Hk1s* in testes from mice sampled at postnatal days (P) 1, 8, 10, 12, 14, 18, 21, 24, 30, 35 and 80, we observed a rapid increase in the *Hk1s* mRNA level starting from P18 and continuing to P35, the period when late pachytene spermatocytes appear and develop through spermiogenesis to produce mature sperm (Fig 1C). These results suggest that HK1S may play roles in late spermatogenesis and/or fertilization.

To more fully understand the physiological function of *Hk1s*, we used the CRISPR/Cas9 technology to generate the *Hk1s* knockout mouse model with targeted deletion of the N-terminal SSR (Fig 1D). We confirmed the successful inactivation of this gene at the genome and mRNA transcription levels (Fig 1E and 1F). Western blot for HK1 antibody (recognizing HK1 and HK1S) showed that HK1(S) exhibited similar expression level between *Hk1s*^+*/*−^ and *Hk1s*^−*/*−^ brain, whereas HK1(S) was absent in *Hk1s*^−*/*−^ testis and sperm (Figs 1G and S1). Our results further show that only HK1S is highly expressed in testis and mature sperm, while HK1 has no expression.

### HK1S is localized on the outer surface of FS

**Fig 2:**
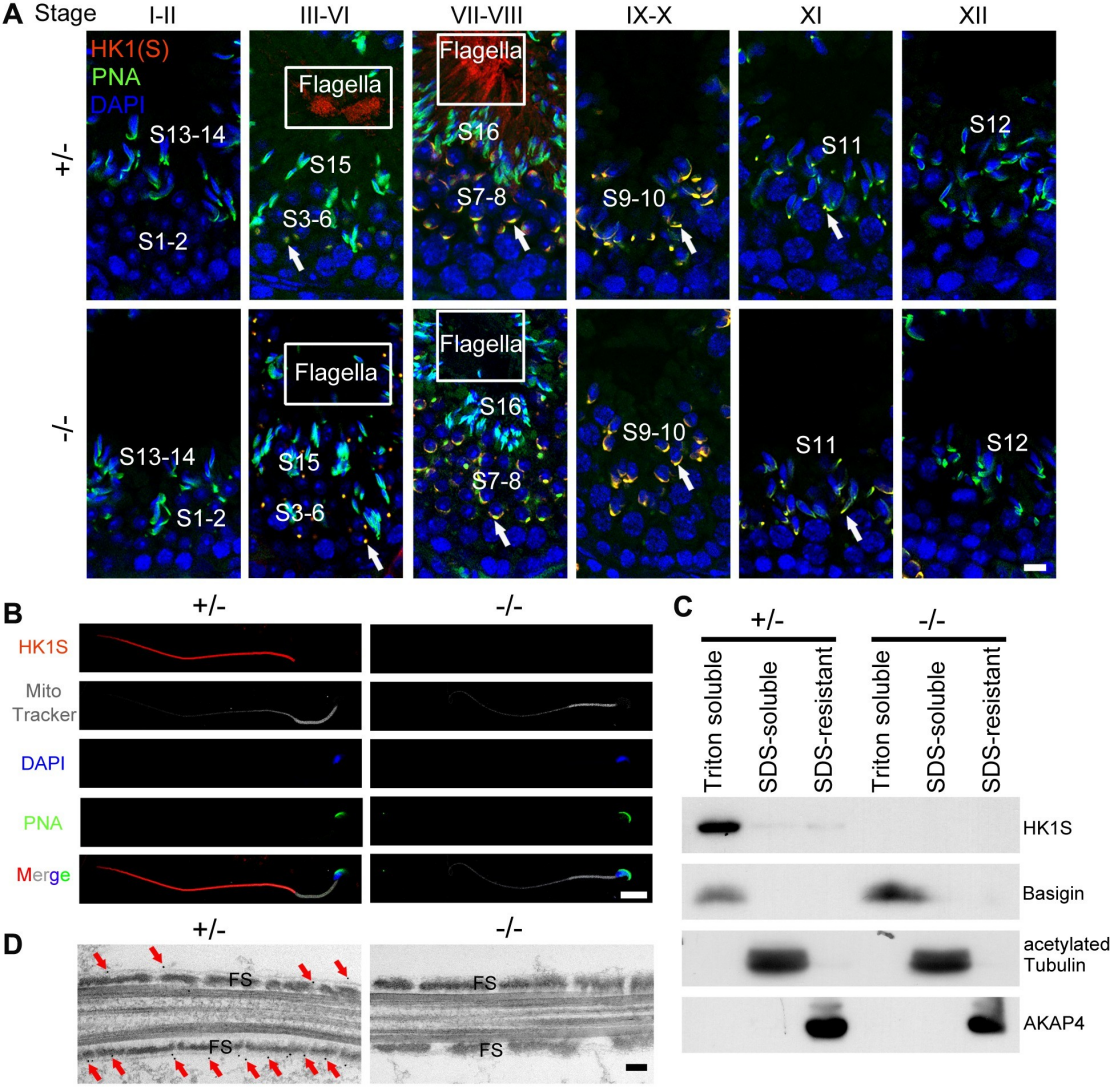
HK1S is localized to the outside of FS. (A) Immunostaining analysis of HK1 and HK1S in *Hk1s*^+*/*−^ and *Hk1s*^−*/*−^ testis sections. The white boxes indicate that HK1S was highly expressed or successfully deleted in the sperm flagella. Arrows indicate nonspecific signaling. Spermatid (S). HK1 and HK1S (red); PNA (green), as the marker of sperm acrosome; DAPI nuclear counterstaining of DNA (blue). Scale bar: 10 μm. (B) Immunostaining analysis of HK1S in *Hk1s*^+*/*−^ and *Hk1s*^−*/*−^ sperm. HK1S (red); MitoTracker staining (white), as the maker for mitochondrial sheath; PNA (green); DAPI (blue). Scale bar: 10 μm. (C) Western blot analysis of *Hk1s*^+*/*−^ and *Hk1s*^−*/*−^ sperm fractionated into Triton X-100 soluble, SDS-soluble, and SDS-resistant insoluble fractions. Basigin, acetylated Tubulin, and AKAP4 were used as makers for Triton-soluble, SDS-soluble, and SDS-resistant fractions, respectively. (D) IEM images of HK1S in the ultrathin sections of mouse sperm. Arrows indicate HK1S gold particles. Fibrous sheath (FS). Scale bars: 100 nm.

Immunofluorescence analysis for HK1 antibody (recognizing HK1 and HK1S, red signal), peanut agglutinin (PNA, acrosome marker) and α-Tubulin (main component of spermatid flagella) showed that the red positive signals were strongly expressed in the acrosomal region of step 3–11 spermatids and flagella of step 15–16 spermatids in the *Hk1s*^+*/*−^ mouse testis, while the red positive signals still remain in the acrosomal regions of step 3–11 spermatids in the *Hk1s*^−*/*−^ mouse testis. This suggests that the fluorescence signals in round spermatids may be false positive, and indicates that the HK1S protein is localized to the flagella region of step 15–16 spermatids (Figs 2A and S2), as has been found in previous studies [8,17]. To confirm this, we also isolated round spermatids and elongating/elongated spermatids from adult testicle samples using STA-PUT velocity sedimentation via bovine serum albumin (BSA) density gradient (S3A Fig) and test HK1(S) expression by western blot. We found that no HK1 or HK1S is expressed in the round spermatids (S3B Fig). Therefore, the above results showed that HK1S was only localized in the flagella region of step 15–16 spermatids in mouse testis. Immunofluorescence staining revealed that HK1S protein was localized in the principal piece of sperm flagellum, which was easily distinguishable by staining the mitochondrial sheath with a MitoTracker dye in *Hk1s*^+*/*−^ sperm. As expected, no HK1S positive signaling was found in *Hk1s*^−*/*−^ sperm (Fig 2B). To further analyze the subcellular localization of HK1S protein, we fractionated sperm proteins into three fractions: the Triton X-100 soluble fraction containing transmembrane and cytosolic proteins, the SDS-soluble fraction containing axonemal proteins, and the SDS-resistant fraction containing proteins localized in the accessory structures such as FS and outer dense fibers [33,34]. Western blot analysis indicated that HK1S was mainly present in the 1% Triton X-100 fraction in *Hk1s*^+*/*−^ sperm (Fig 2C), which was consistent with previous study [23]. Electron microscopic immunocytochemical staining further revealed that gold particles were localized on the outer surface of FS of *Hk1s*^+*/*−^ sperm, while *Hk1s*^−*/*−^ sperm section had no visible gold particles (Fig 2D). These data indicated that HK1S is localized on the outer surface of FS, and it may play important roles in mature sperm.

### *Hk1s*^−/−^ male mice are infertile but have normal sperm morphology

**Fig 3:**
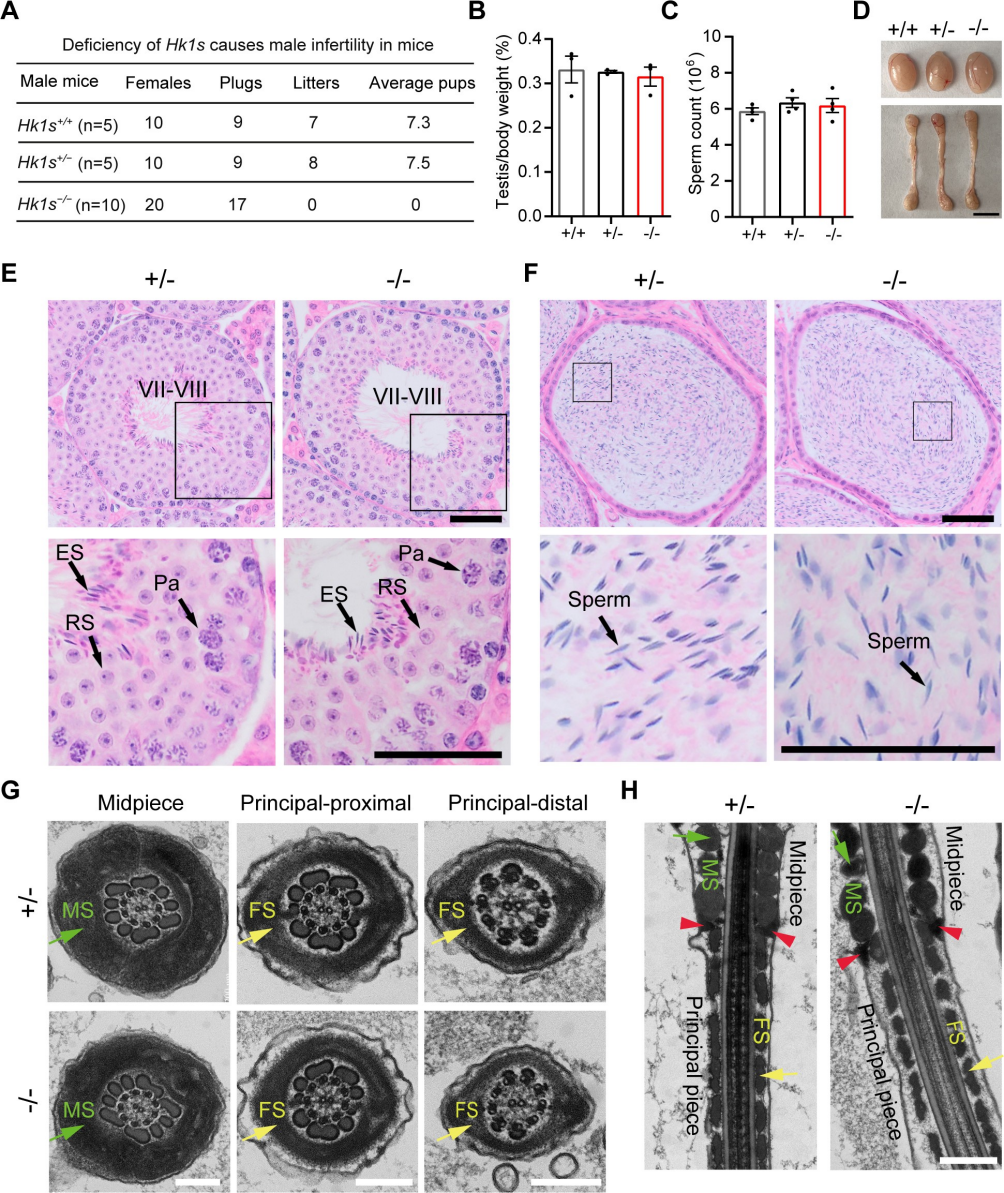
*Hk1s*^−/−^ male mice are infertile but have normal sperm morphology. (A) Number of litters born per plug detected. (B) Ratios of testis weight to body weight of *Hk1s*^+*/*+^, *Hk1s*^+*/*−^ and *Hk1s*^−*/*−^ mice. Mice number (n = 3) per genotype. (C) The number of sperm in cauda epididymides of *Hk1s*^+*/*+^, *Hk1s*^+*/*−^ or *Hk1s*^−*/*−^ male mice was counted and analyzed. Mice number (n = 4) per genotype. (D) Representative images showing the morphology of testes and epididymides in adult *Hk1s*^+*/*+^, *Hk1s*^+*/*−^, or *Hk1s*^−*/*−^ mice. Scale bars: 5 mm. (E) H&E staining in testis sections of *Hk1s*^+*/*−^ and *Hk1s*^−*/*−^ mice. Pa, pachytene; Rs, round spermatids; ES, elongated spermatids. Scale bars: 50 μm. (F) H&E staining in cauda epididymis sections of *Hk1s*^+*/*−^ and *Hk1s*^−*/*−^ mice. Scale bars: 50 μm. (G) Ultrastructural analysis of cross-section of sperm from the cauda epididymis obtained from *Hk1s*^+*/*−^ and *Hk1s*^−*/*−^ mice using TEM. MS, mitochondrial sheath (green arrow); FS, fibrous sheath (yellow arrow). Scale bars: 200 nm. (H) Ultrastructural analysis of longitudinal section near the annulus of sperm from the cauda epididymis obtained from *Hk1s*^+*/*−^ and *Hk1s*^−*/*−^ mice using TEM. MS, mitochondrial sheath (green arrow); FS, fibrous sheath (yellow arrow); Red arrowheads indicate the sperm annulus. Scale bars: 500 nm.

To examine whether HK1S is required for fertility, individual *Hk1s*^+*/*+^, *Hk1s*^+*/*−^, and *Hk1s*^−*/*−^ male mice were caged with wild-type females over a period of two months. The fertility of *Hk1s*^+*/*−^ male mice was comparable to that of *Hk1s*^+*/*+^ controls, but *Hk1s*^−*/*−^ adult male mice were infertile. They mated with wild-type female mice (which formed normal vaginal plugs), but did not produce any offspring (Fig 3A). Conversely, *Hk1s*^−*/*−^ females were fertile when they were mated with *Hk1s*^+*/*+^ or *Hk1s*^+*/*−^ male mice. These results demonstrate that HK1S is essential for male fertility.

We next investigated potential mechanisms related to the infertility of *Hk1s*^−*/*−^ male mice, by carefully monitoring the development of *Hk1s*^+*/*+^, *Hk1s*^+*/*−^, and *Hk1s*^−*/*−^ male mice. On reaching sexual maturity, *Hk1s*^+*/*+^, *Hk1s*^+*/*−^ and *Hk1s*^−*/*−^ littermates had similar testis to body weight ratio, sperm densities in cauda epididymides, and gross morphology (Fig 3B–3D). Moreover, there were no obvious discrepancies in the histology and germ cell apoptosis of *Hk1s*^+*/*−^ and *Hk1s*^−*/*−^ testes or cauda epididymides (Figs 3E–3F and S4). A detailed investigation based on transmission electron microscopy (TEM) showed no obvious defects in the ultrastructures of *Hk1s*^+*/*−^ versus *Hk1s*^−*/*−^ sperm, as both genotypes exhibited normal ultrastructural features of the midpiece and principal piece (proximal and distal regions) (Fig 3G and 3H). Collectively, these observations strongly suggest that HK1S is not required for spermatogenesis and sperm production.

### *Hk1s* deficiency reduces sperm glycolytic metabolism but does not affect sperm motility and ATP levels

**Fig 4:**
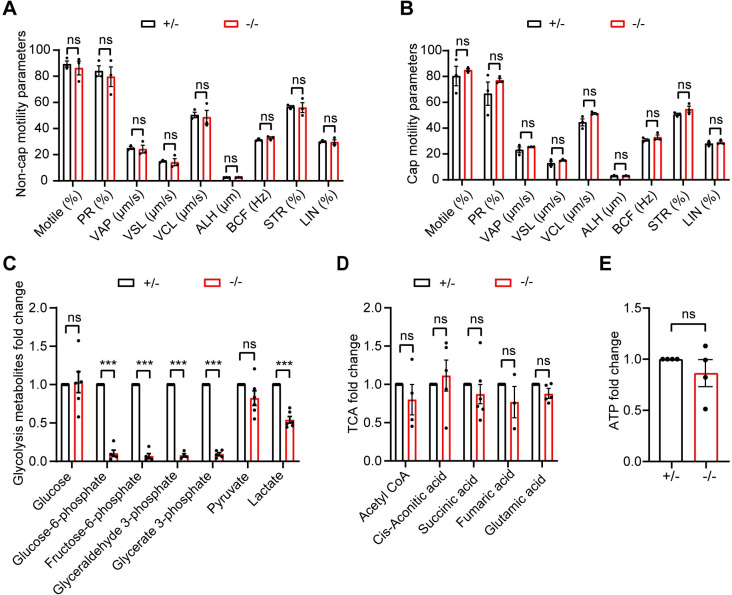
*Hk1s* deficiency reduces sperm glycolytic metabolism but does not affect sperm motility and ATP levels. (A-B) A CASA system was used to measure sperm motility parameters for 10 min (non-capacitated condition, Non-cap) and 2 h (capacitated condition, Cap) in TYH medium. PR, progressive motility; VAP, average path velocity; VSL, straight line velocity; VCL, curvilinear velocity; ALH, amplitude of lateral head displacement; BCF, beat cross frequency; STR, straightness; LIN, Linearity. Mice number (n = 3) per genotype. Error bars: SEM. Statistics, Student’s *t*-Test. ns: non-significant. (C-D) Changes of glycolysis and TAC metabolites in the capacitated *Hk1s*^−*/*−^ sperm compared with control by LC-MS-based targeted metabolomics analysis. Mice number (n = 3–6) per genotype. Error bars: SEM. Statistics, Student’s *t*-Test. ***, *P* < 0.001; ns: non-significant. (E) Sperm ATP levels in the capacitated *Hk1s*^−*/*−^ sperm compared with control by LC-MS-based targeted metabolomics analysis. Mice number (n = 4) per genotype. Error bars: SEM. Statistics, Student’s *t*-Test. ns: non-significant.

To clarify the cause of impaired male fertility of *Hk1s* mutant mice, sperm motility was examined by real-time videos and computer-assisted sperm analysis (CASA) system. Our results found that *Hk1s*^−*/*−^ mice showed normal sperm motility parameters in the non-capacitated conditions (Fig 4A, S1 and S2 Movies) and capacitated conditions (Fig 4B, S3 and S4 Movies). HK1S catalyzes the first step in committed step of glycolysis, therefore, we employed targeted liquid chromatography-mass spectrometry (LC-MS)-based metabolomics analysis to explore the two major energy metabolic pathways of sperm-glycolysis and tricarboxylic acid cycle (TAC) in the capacitated *Hk1s*^+*/*−^ and *Hk1s*^−*/*−^ sperm. Compared to *Hk1s*^+*/*−^ sperm, glucose and pyruvate were no significant difference in *Hk1s*^−*/*−^ sperm, due to the presence of these components in the medium (Fig 4C). However, the other intermediate metabolites involved in glycolysis were significantly downregulated (Fig 4C). In addition, TCA cycle showed no substantial alterations (Fig 4D) in *Hk1s*^−*/*−^ sperm. Interestingly and surprisingly, the total ATP production showed no obvious difference (Fig 4E). We further utilized CellTiter-Glo Luminescent Cell Viability Assay to monitor sperm ATP levels in the non-capacitated and capacitated conditions, and found similar results (S5 Fig). Collectively, these data suggest that the production of ATP in sperm may be achieved via pathways other than glycolysis and OXPHOS.

### Disruption of *Hk1s* leads to failure of sperm-ZP penetration

**Fig 5:**
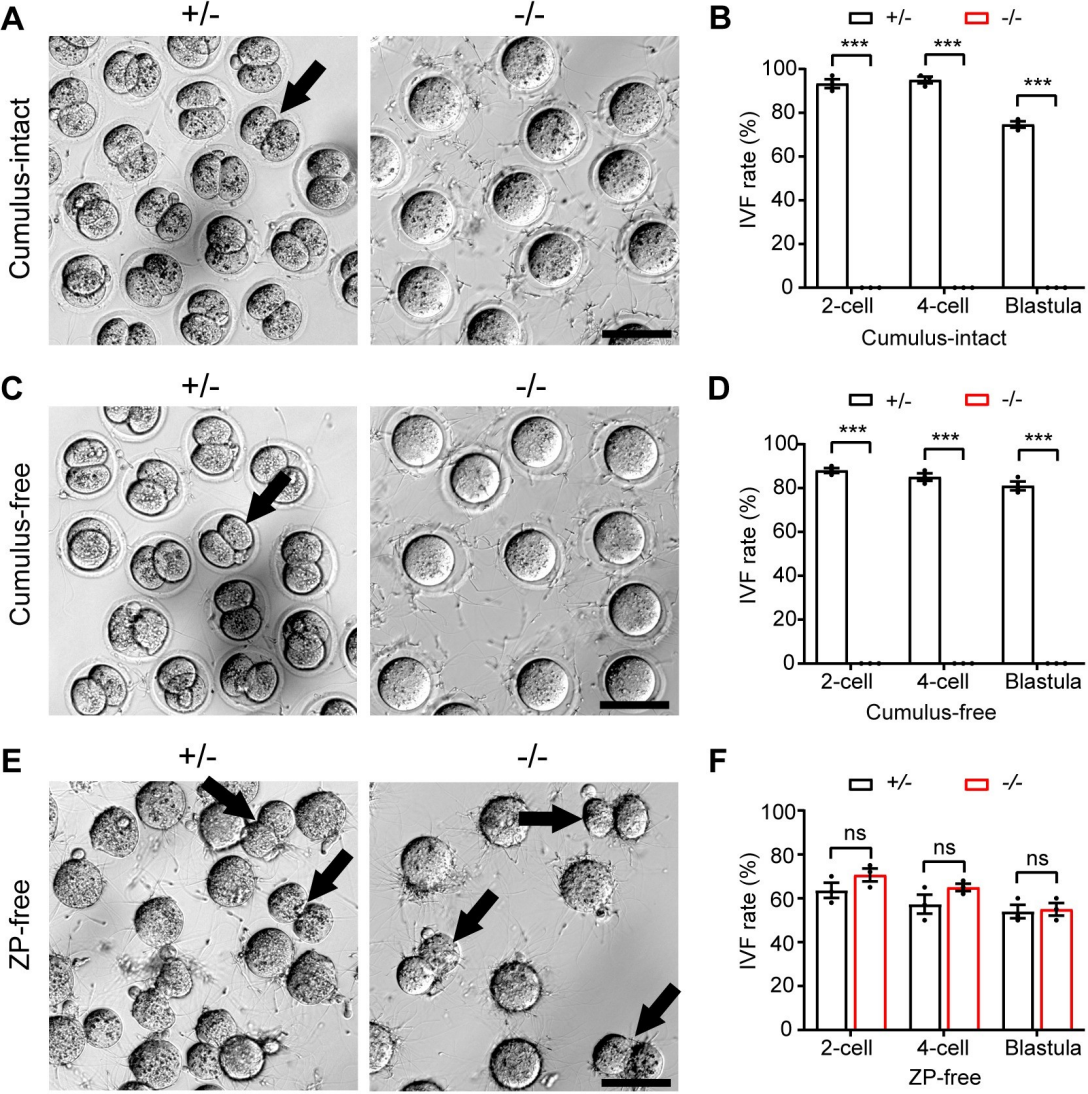
*In vitro* fertility of *Hk1s*^−/−^ male mice. (A) Representative images of IVF with cumulus-intact oocytes. Oocytes were collected from oviducts of wild-type female and co-cultured with *Hk1s*^+*/*−^ and *Hk1s*^−*/*−^ sperm for 24 h. Scale bar: 100 μm. Arrow indicates 2-cells. (B) Development of 2-cell, 4-cell, and blastula embryo ratio of IVF with cumulus-intact oocytes between *Hk1s*^+*/*−^ and *Hk1s*^−*/*−^ groups. Mice number (n = 3) per genotype. Error bars: SEM. Statistics, Student’s *t*-Test. ***, *P* < 0.001. (C) Representative images of IVF with cumulus-free oocytes. Scale bar: 100 μm. Arrow indicates 2-cell. (D) Development of 2-cell, 4-cell, and blastula embryo ratio of IVF with cumulus-free oocytes between *Hk1s*^+*/*−^ and *Hk1s*^−*/*−^ groups. Mice number (n = 3) per genotype. Error bars: SEM. Statistics, Student’s *t*-Test. ***, *P* < 0.001. (E) Representative images of IVF with ZP-free oocytes. Scale bar: 100 μm. Arrows indicate 2-cells. (F) Development of 2-cell, 4-cell, and blastula embryo ratio of IVF with ZP-free oocytes between *Hk1s*^+*/*−^ and *Hk1s*^−*/*−^ groups. Mice number (n = 3) per genotype. Error bars: SEM. Statistics, Student’s *t*-Test. ns: non-significant.

In order to identify the specific stage at which sperm encounter difficulties during fertilization, we performed IVF. Approximately 90% of 2-cells were found after 24 h in the *Hk1s*^+*/*−^ sperm with cumulus-intact oocytes, and 2-cells could develop to the blastocyst stage. However, no 2-cells were found in the *Hk1s*^−*/*−^ sperm, and fertilization rate was zero (Fig 5A and 5B). In addition, both *Hk1s*^+*/*−^ and *Hk1s*^−*/*−^ sperm could penetrate cumulus cell layers with cumulus-intact oocytes (S6A Fig), suggesting that *Hk1s*^−*/*−^ sperm could penetrate the cumulus cell layers, but not fertilize cumulus-intact oocytes. Removing cumulus (cumulus-free) oocytes could not rescue impaired fertilization rates (Fig 5C and 5D). However, the fertilization rate of *Hk1s*^−*/*−^ sperm with ZP-free oocytes was comparable to that of the control, and 2-cells could develop to the blastocyst stage at a similar ratio between *Hk1s*^+*/*−^ and *Hk1s*^−*/*−^ groups (Fig 5E and 5F). These findings indicate that knockout of *Hk1s* impairs the ability of sperm to bind or penetrate the ZP but not sperm-oocyte fusion. We thus examined the ZP-binding ability of sperm using cumulus-free oocytes and found that *Hk1s*^−*/*−^ sperm showed normal ZP-binding ability compared with wild-type sperm (S6B Fig). These results suggest that *Hk1s*^−*/*−^ sperm are defective in the ZP penetration, and lead to severe fertility defects *in vivo*.

### Disruption of *Hk1s* affects the sperm migration into oviduct and reduces acrosome reaction

**Fig 6:**
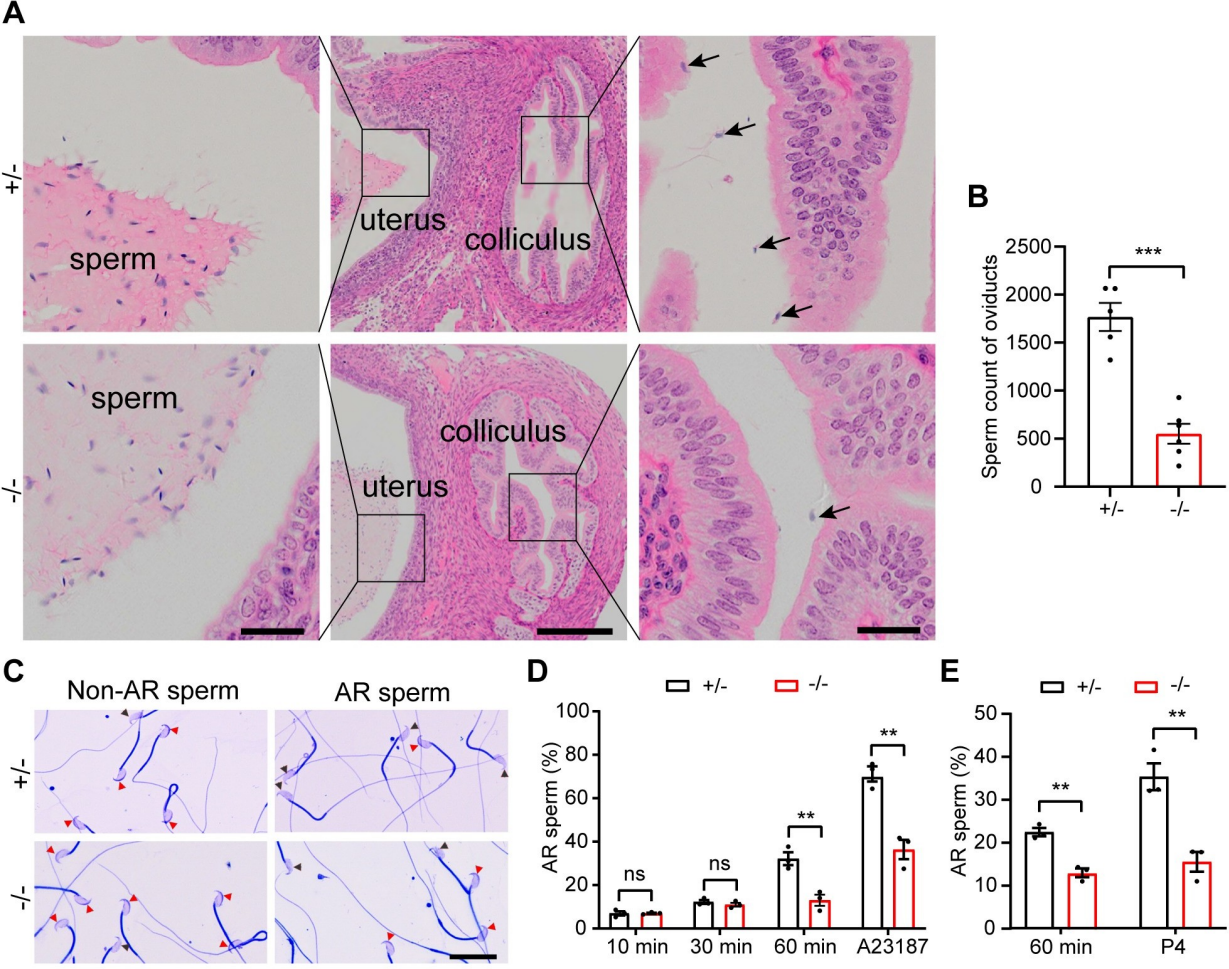
Disruption of *Hk1s* affects the sperm migration into oviduct and reduces acrosome reaction. (A) H&E staining showed the migration of sperm from uterus into oviducts. Uterus, colliculus of the UTJ, and sperm ejaculated into uterus are shown in the middle panels. Left and right panel insets show higher magnification. Arrows show sperm. More than three females plugged by a male of each genotype were detected and representative images are shown. Left and right bar: 20 μm, middle bar: 200 μm. (B) Sperm number in the bilateral oviducts of plugged females were counted and analyzed. Mice number (n = 5–6) per genotype. Error bars: SEM. Statistics, Student’s *t*-Test. ***, *P* < 0.001. (C) Non-AR and AR sperm were stained by Coomassie Brilliant Blue R-250, and acrosome disappeared in the AR sperm. Black arrowheads indicate the disappeared acrosome; Red arrowheads indicate the intact acrosome. Scale bar: 20 μm. (D) The percentage of the AR in *Hk1s*^+*/*−^ and *Hk1s*^−*/*−^ sperm, which was incubated after 10 min, 30 min, 60 min in the capacitating medium. Calcium ionophore A23187 was added to induce the AR for 60 min. Mice number (n = 3) per genotype. Error bars: SEM. Statistics, Student’s *t*-Test. **, *P* < 0.01; ns: non-significant. (E) The percentage of the AR was induced by progesterone (P4) in capacitated *Hk1s*^+*/*−^ and *Hk1s*^−*/*−^ sperm. Mice number (n = 3) per genotype. Error bars: SEM. Statistics, Student’s *t*-Test. **, *P* < 0.01.

Ejaculated sperm need to migrate from the uterus to oviducts and reach the oocytes to complete fertilization. To determine whether infertility was caused by impaired migration of the *Hk1s*^−*/*−^ sperm in the oviduct, we analyzed sperm that arrived at oviducts of wild-type females plugged by *Hk1s*^+*/*−^ or *Hk1s*^−*/*−^ male mice. Histological analysis showed that *Hk1s*^−*/*−^ sperms were much less in the colliculus of the utero-tubal junction (UTJ) 2 h after copulation, while there were numerous sperm in the nearby uterine lumen (Fig 6A). The count of all sperm in the oviduct showed that the number of *Hk1s*^−*/*−^ sperm was less than that of control (control: 1767.2 vs *Hk1s*^−*/*−^: 551.0) (Fig 6B). These results suggest that disruption of *Hk1s* affects the sperm migration from the uterus to the oviduct.

It is widely accepted that sperm must undergo acrosome reaction to penetrate the ZP of oocyte, therefore, we analyzed the percentage of acrosome-reacted sperm in the capacitating medium from *Hk1s*^−*/*−^ sperm. We found that the percentage of acrosome-reacted sperm did not have a significant difference between the *Hk1s*^+*/*−^ and *Hk1s*^−*/*−^ sperm after incubation for 10 min and 30 min. After 60 min of incubation, the percentage was significantly lower than in *Hk1s*^+*/*−^ sperm (control: 32.2% vs *Hk1s*^−*/*−^: 13.0%), even after A23187 treatment, it could not reach the same level as the control group (control: 71.2% vs *Hk1s*^−*/*−^: 36.4%) (Fig 6C and 6D). The results were consistent with the progesterone-induced sperm acrosome reaction (control: 45.7% vs *Hk1s*^−*/*−^: 26.0%) (Fig 6E). These data indicate that HK1S is required for the acrosome reaction.

### *Hk1s* deficiency disrupts tyrosine phosphorylation associated with sperm capacitation

**Fig 7:**
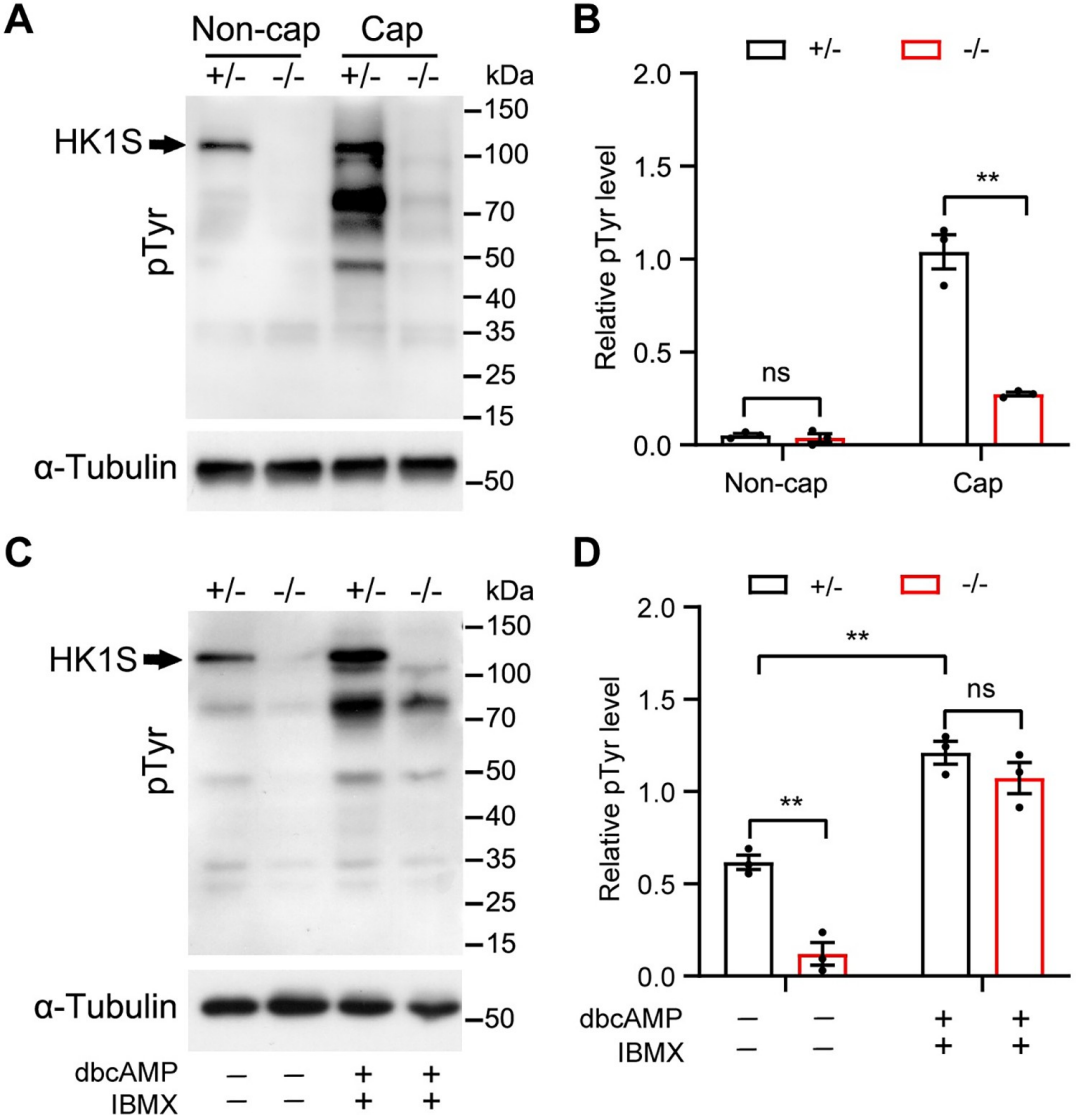
HK1S is required for protein tyrosine phosphorylation associated with sperm capacitation. (A) Western blot analysis for phosphotyrosine (pTyr) of *Hk1s*^+*/*−^ and *Hk1s*^−*/*−^ sperm under non-capacitating (Non-cap) and capacitating (Cap) medium for 60 min. α-Tubulin was used as a loading control. Arrow indicates HK1S, which was a tyrosine phosphorylated form. (B) The quantification of protein pTyr level in *Hk1s*^+*/*−^ and *Hk1s*^−*/*−^ sperm under non-capacitation (Non-cap) and capacitation (Cap) medium for 60 min. Mice number (n = 3) per genotype. Error bars: SEM. Statistics, Student’s *t*-Test. **, *P* < 0.01; ns: non-significant. (C) Western blot analysis for pTyr of *Hk1s*^+*/*−^ and *Hk1s*^−*/*−^ sperm under capacitating medium ±1 mM dbcAMP and 100 μM IBMX for 60 min. α-Tubulin was used as a loading control. Arrow indicates HK1S. (D) The quantification of protein pTyr level in *Hk1s*^+*/*−^ and *Hk1s*^−*/*−^ sperm under capacitating medium ±1 mM dbcAMP and 100 μM IBMX for 60 min. Mice number (n = 3) per genotype. Error bars: SEM. Statistics, Student’s *t*-Test. **, *P* < 0.01; ns: non-significant.

Capacitation is the biochemical process that sperm undergo after ejaculation into the female reproductive tract and is thought to be a prerequisite for the acrosome reaction [35,36]. An increase in the level of protein tyrosine phosphorylation is an important aspect of sperm capacitation [27,37], therefore we monitored the level of tyrosine phosphorylation by western blot. In the non-capacitated condition, the level of tyrosine phosphorylation was comparable between *Hk1s*^+*/*−^ and *Hk1s*^−*/*−^ sperm. However, in the capacitated condition, we noted that the level of protein tyrosine phosphorylation in *Hk1s*^−*/*−^ sperm was significantly decreased compared with *Hk1s*^+*/*−^ sperm (Fig 7A and 7B), suggesting that defects in protein tyrosine phosphorylation may contribute to the failure of acrosome reaction. In addition, HK1S was a tyrosine phosphorylated form of hexokinase [38,39], which was further confirmed by our results in the *Hk1s*^+*/*−^ and *Hk1s*^−*/*−^ sperm (Fig 7A and 7C, arrow).

Cyclic AMP analogs that activate protein kinase A (PKA) stimulate the same pattern of tyrosine phosphorylation at an accelerated rate [40,41]. To determine if the PKA signal transduction pathway in sperm from *Hk1s*^−*/*−^ mice is responsive to capacitation cues, we monitored protein tyrosine phosphorylation in the presence of a cAMP analog dibutyryl-cAMP (dbcAMP) and a phosphodiesterase inhibitor 3-isobutyl-1-methylxanthine (IBMX) in the capacitated condition. In agreement with previous reports [41], protein tyrosine phosphorylation was exacerbated by exposure of sperm to dbcAMP and IBMX in *Hk1s*^+*/*−^ sperm (Fig 7C). In addition, a significant increase in tyrosine phosphorylation was also observed when sperm were exposed to dbcAMP and IBMX in *Hk1s*^−*/*−^ sperm, and it could fully recover to the same level as the control group (Fig 7D). Taken together, these results suggest that HK1S can trigger protein tyrosine phosphorylation by mediating cAMP-PKA pathway.

## Discussion

HK1S, one of the spermatogenic cell-specific variant isozymes that was reported to be specifically present in the mature sperm, has been localized to the principal piece in mice and has a novel spermatogenic cell-specific region at the N-terminus [8]. Similarly, our result showed that HK1S was restricted to the flagella region of step 15–16 spermatids in mouse testis, and localized in the principal piece of sperm flagellum. In addition, HK1S in sperm was mainly solubilized with 1% Triton X-100 fraction, consistent with a previous report [23]. Moreover, our more detailed observations found that HK1S was localized on the outer surface of the FS; unlike GAPDHS, ENO4, and other sperm-specific aldolase isozymes that are tightly bound to the FS [18,20,42].

In the present study, we have shown that HK1S is essential for male fertility. The histological results in the testis and cauda epididymis of *Hk1s*^−*/*−^ mice indicated that the spermatogenesis process is normal. In addition, various parameters of mature sperm in epididymis including the ultrastructures from the TEM assay all showed no difference between *Hk1s*^−*/*−^ and *Hk1s*^+*/*−^ male mice, suggesting that this isozyme is not required for spermatogenesis and sperm morphogenesis. Among enzymes involved in the glycolytic pathway, previous studies have shown that GAPDHS [30], PGK2 [32], and LDHC [31] are essential for male fertility and knockout of them respectively does not disrupt sperm structure, which is similar to the phenotypes of knocking out *Hk1s*.

Glycolysis is the main source of ATP in sperm of mice and, presumably, of many other mammalian species to provide the energy required for the flagellar activity that produces sperm motility [16]. The targeted disruption of *Gapdhs* [30], *Pgk2* [32], or *Ldhc* [31] in male mice severely reduced sperm motility and ATP levels. Our results show that the detailed intermediates involved in glycolysis are significantly downregulated in *Hk1s*^−*/*−^ sperm. In contrast, *Hk1s*^−*/*−^ sperm have normal motility and ATP levels. Amara et al. found other metabolic pathways such as fatty acid β-oxidation (FAO) by proteomic studies of human sperm may also contribute to the production of energy [43]. Moreover, treating the stallion sperm with carnitine palmitoyl transferase 1, a rate-limiting enzyme of β-oxidation, can lead to decreased sperm motility [44]. Knockout of *Slc22a14* disrupts sperm FAO activity and leads to male infertility in mice [14]. Collectively, the production of ATP in the male gamete may be achieved via pathways other than glycolysis and OXPHOS in *Hk1s*^−*/*−^ sperm.

To further seek the cause inducing the infertility of *Hk1s*^−*/*−^ male mice, IVF was performed. We found that *Hk1s*^−*/*−^ sperm were unable to fertilize cumulus-intact or cumulus-free oocytes, but they were able to fertilize ZP-free oocytes at a similar ratio comparable to *Hk1s*^+*/*−^ sperm. On the other hand, *Gapdhs*^−*/*−^, *Pgk2*^−*/*−^, or *Ldhc*^−*/*−^ sperm showed low fertilization rates (<10%) when used in IVF assays with cumulus-oocyte complexes [31,41]. However, when the ZP was removed or drilled, the number of these oocytes fertilized increased by one-third observed with controls, suggesting that it implies a defect in sperm-egg fusion by *Gapdhs*^−*/*−^, *Pgk2*^−*/*−^ or *Ldhc*^−*/*−^ sperm. These discrepancies may be related to the normal sperm motility and ATP levels in the *Hk1s*^−*/*−^ sperm. In addition, *Hk1s*^−*/*−^ sperm did not undergo the protein tyrosine phosphorylation changes characteristic of capacitation, which was similar to *Gapdhs*^−*/*−^, *Pgk2*^−*/*−^ or *Ldhc*^−*/*−^ sperm [31,41]. Cyclic AMP analogs that activate PKA stimulate the same pattern of tyrosine phosphorylation at an accelerated rate [41]. The level of the protein tyrosine phosphorylation had a significant increase in *Hk1s*^−*/*−^ sperm exposed to dbcAMP and IBMX, which promotes tyrosine phosphorylation under normal conditions by stimulating PKA and inhibiting phospho-diesterases. In contrast, *Gapdhs*^−*/*−^ or *Pgk2*^−*/*−^ sperm did not show an increase the tyrosine phosphorylation in capacitating medium with dbcAMP and IBMX [41]. These results suggest that the pathway mediating PKA triggering of tyrosine phosphorylation is responsive in *Hk1s*^−*/*−^ sperm, but is unresponsive in *Gapdhs*^−*/*−^ or *Pgk2*^−*/*−^ sperm.

According to a previous study, the release of PKA from A-Kinase anchoring protein (AKAP) in capacitated sperm promotes a sudden Ca^2+^ influx that starts in the principal piece, and propagates to the head triggering the acrosome reaction [45]. In our study, we found that HK1S is localized on the outer surface of FS and not expressed in the acrosome, but the percentage of *Hk1s*^−*/*−^ acrosome-reacted sperm had a significantly lower not only after 60 min of incubation in the capacitating medium but also after physiological agonists treatment, such as calcium ionophore A23187 or progesterone, compared to the control group. These results suggest that defects in the cAMP-PKA pathway may impact the AR and contribute to the failure to fertilize ZP-intact oocytes. On the other hand, *Gapdhs*^−*/*−^ or *Pgk2*^−*/*−^ sperm did not show defects in ionophore-induced AR, however, their sperm motility and ATP levels were markedly reduced. This indicates defects in energy metabolism may contribute to the failure of *Gapdhs*^−*/*−^ or *Pgk2*^−*/*−^ sperm to fertilize ZP-intact oocytes.

In summary, our study demonstrated that HK1S plays a crucial role in male fertility in mice. Its deletion leads to defects in the number of sperm entering the oviduct, acrosome reaction, cAMP-PKA pathway, and protein tyrosine phosphorylation. Although the levels of sperm motility and ATP were relatively normal in *Hk1s*^*-/-*^ sperm, the detailed intermediates involved in glycolysis were significantly downregulated, indicating that the HK1S is essential for mouse sperm glycolysis. Furthermore, three unique human type 1 hexokinase mRNAs exhibit testis-enriched expression [46], and these functions may be conserved in humans as well. By identifying HK1S-associated molecules and their functions, we may gain a better understanding of the causes of human male infertility.

## Materials and methods

### Ethics statement

All animal experiments were approved by the Chinese Ministry of Health national guidelines, and performed in accordance with institutional regulations of the Institutional Animal Care and Use Committee at the National Institute of Biological Sciences, Beijing (#SYXK [jing] 2023–0007).

### Animals

All mice in this study were C57BL/6 strains. Mice were housed in the National Institute of Biological Sciences, Beijing. The gene-modified mice generated in this study were available through Transgenic Animal Center, National Institute of Biological Sciences, Beijing.

### Generation of *Hk1s* knockout mice

*Hk1s*^−*/*−^ mice were generated using the CRISPR/Cas9 technology. The sgRNAs were prepared using MEGAshortscript T7 Transcription kit (AM1354, Ambion, USA) according to the manufacturer’s instructions. DNA fragments containing the coding exon 3–4 of the *Hk1s* (N-terminal SSR) and two homology arms were used as donor templates. After co-incubation of Cas9 protein and sgRNAs, the Cas9-sgRNA complex and donor templates were injected into C57BL/6 zygotes. Injected zygotes were transferred into pseudopregnant CD1 female mice (20–30 zygotes per pseudopregnant mice). The targeting strategy, including the sgRNA sequences and the knockout alleles obtained, are depicted in Fig 1D. The primers used for genotyping were listed in the S1 Table.

### Fertility assessment

Adult male mice (*Hk1s*^+*/*+^, *Hk1s*^+*/*−^, *Hk1s*^−*/*−^) were caged for 2 months with wild-type females. Copulation was confirmed by checking for vaginal plugs. The number of litters and the average number of progeny per litter were analyzed.

### Histological analysis

Standard Hematoxylin and Eosin (H&E) staining was performed on paraffin sections as described previously [47]. Testes and cauda epididymides were freshly fixed in Davidson’s fixative solution (Formaldehyde: Ethanol: Glacial acetic acid: H_2_O = 6: 3: 1: 10) overnight at 4°C. Samples were dehydrated in an ethanol series (70%, 80%, 90%, 100%), and embedded in paraffin. The 5 μm thickness sections were cut using a microtome (RM2245, Leica, Germany) and mounted on adhesion microscope slides. After re-hydration, the slides were stained with H&E following standard protocols. Images were acquired using Olympus VS120 microscope.

### Isolation of spermatids using STA-PUT velocity sedimentation

Round spermatids and elongating/elongated spermatids were isolated from adult *Hk1s*^+*/*−^ or *Hk1s*^−*/*−^ testes using STA-PUT velocity sedimentation according to previous study [48]. Briefly, the seminiferous tubules were digested in 10 ml Krebs (KH_2_PO_4_ 2 mM, NaCl 119.7 mM, MgSO_4_·7H_2_O 1.2 mM, dextrose 13.9 mM, CaCl_2_·2H_2_O 1.3 mM, KCl 4.8 mM, NaHCO_3_ 25.2 mM) with 1 mg/ml collagenase IV (40510ES60, Yeasen, China) and 0.2 mg/ml DNase I (10608ES80, Yeasen, China) at 34°C for 20 min. Then tubules were washed with 5 ml Krebs twice and were subsequently digested with 10 ml 0.05% trypsin (25300–062, Gibco, USA) containing 0.3 mg/ml DNase I at 34°C for 8 min to prepare single-cell suspensions. Enzymatic digestion was quenched with 1 ml of 10% fetal bovine serum (FBS) (164210, Procell, China). Cell suspensions were sieved through a 40 μm cell strainer (352340, BD Falcon, USA). The cell pellet was collected by centrifugation at 2500 rpm for 5 min at 4°C and resuspended in 3 ml Krebs containing 0.5% BSA. To prepare 1–3% linear BSA gradient, add 5 ml of 1% BSA solution to the bottom of the tube and gradually slowly add 5 ml of 2% and 3% BSA solution to the bottom of the tube. Then, cell suspension was gently loaded onto a 1–3% linear BSA gradient and separated by sedimentation velocity at unit gravity for 1.5 h on ice. Enriched germ cell fractions were carefully collected per tube, starting from the top of the BSA gradient. Number the tubes in the same order as the fractions collected (No.1-8). No.1-3 and No.5-8 cell fractions were respectively pooled together. The purities of these two cell samples were evaluated and identified by their morphological characterization and immunofluorescence analysis with DAPI and PNA.

### Western blot

Testes and sperm were lysed in 1% Triton X-100 lysis buffer (50 mM NaCl, 20 mM Tris·HCl, pH 7.5) with 1: 10 protease inhibitor cocktail (#04693116001, Roche, Switzerland). Homogenized lysates were rotated for 60 min at 4°C, and centrifuged at 13000 rpm for 20 min at 4°C. The protein concentrate of supernatants was determined using bicinchoninic acid (BCA) protein assay (#23225, Thermo Fisher Scientific, USA). The equal quality of proteins of each sample was electrophoresed on sodium dodecyl sulfate-polyacrylamide gel electrophoresis (SDS-PAGE) and transferred to polyvinylidene difluoride (PVDF) membranes (IPVH00010, MilliporeSigma, USA). The membrane was blocked with 5% skim milk in TBST (Tris-buffered saline containing 0.1% Tween-20) for 2 h at room temperature, and respectively incubated with primary antibodies diluted in antibody diluent (WB500D, NCM Biotech, China) at 4°C overnight. The PVDF membrane was then washed three times in 0.1% Tween-20 in TBST and incubated with secondary antibodies for 60 min at room temperature. After washing with TBST, the membrane was treated with the Pierce ECL 2 western blot Substrate (#34577, Thermo Fisher Scientific, USA), and signals were detected by XBT X-ray film.

The primary antibodies used are as follows: rabbit anti-HK1 (C35C4) (1: 2000, #2024, Cell Signaling Technology, USA), mouse anti-phosphotyrosine clone 4G10 (1: 1000, #05–321, MilliporeSigma, USA), mouse anti-Basigin (1: 2000, sc-46700, Santa Cruz biotechnology, USA), mouse anti-acetylated Tubulin (1: 2000, T7451, Sigma-Aldrich, USA), mouse anti-AKAP4 (1: 2000, sc-135827, Santa Cruz biotechnology, USA), mouse anti-α-Tubulin (1: 1000, AC012, Abclonal Technology, China). The secondary antibodies used are as follows: horseradish peroxidase-conjugated goat anti-rabbit IgG (1: 5000, A6154, MilliporeSigma, USA) and horseradish peroxidase-conjugated goat anti-mouse IgG (1: 5000, A4416, MilliporeSigma, USA)

### Protein tyrosine phosphorylation assay

The sperm isolated from cauda epididymis were suspended in 500 μl of capacitating medium (TYH, 119.3 mM NaCl, 4.7 mM KCl, 1.2 mM KH_2_PO_4_, 1.2 mM MgSO_4_, 5.6 mM glucose, 0.5 mM sodium pyruvate, 1.7 mM CaCl_2_, and 20 mM HEPES, 15 mM NaHCO_3_ and 5 mg/ml BSA) or non-capacitating medium (NaHCO_3_-free and BSA-free TYH) during 60 min at 37°C in 5% CO_2_. Alternatively, incubation was carried out in the capacitating media containing the following compounds: 1 mM dbcAMP (D0627, Sigma-Aldrich, USA) and 100 μM IBMX (I5879, Sigma-Aldrich, USA). After centrifugation at 3000 rpm for 5 min, the pellet was resuspended in 100 μl lysis buffer (P0013, Beyotime, China) and mixed well. Then, lysis was sonicated on ice prior to boiling and centrifugation to yield a soluble fraction. Protein extracts from sperm suspension were subjected to anti-phosphotyrosine immunoblot analysis. For each experiment (n = 3), the pTyr blots were analyzed using ImageJ software. For comparison between blots, pixels for each lane contained in the region (proteins in the 40–100 kDa range for pTyr) were quantified and normalized using the α-Tubulin.

### Sperm motility analysis

For analysis of motility, sperm were isolated from cauda epididymis and incubated for 10 min (non-capacitated condition) and 2 h (capacitated condition) in TYH medium at 37°C in 5% CO_2_. Real-time videos were recorded immediately after isolation, at 10 min and 2 h after isolation using Hoffman Modulation Contrast microscope (OLYMPUS IX71). A CASA system (Version.14 CEROS, Hamilton Thorne Research, USA) with a Slide Warmer (#720230, Hamilton Thorne Research, USA) was used to quantify parameters of sperm motility with the following settings: minimal contrast, 30; minimal cell size, 4 pixels; and 30 frames were acquired at a frame rate of 60 Hz.

### Sperm protein fractionation

The sperm were suspended in 1% Triton X-100 lysis buffer (50 mM NaCl, 20 mM Tris·HCl, pH 7.5 with protease inhibitor cocktail) and incubated at 4°C for 60 min. The sample was centrifuged at 13000 rpm for 10 min to separate the Triton-soluble fraction (supernatant) and the Triton-resistant fraction (pellet). The pellet was resuspended in 1% SDS lysis buffer (75 mM NaCl, 24 mM EDTA, pH 6.0) and incubated at room temperature for 60 min. The sample was centrifuged at 13000 rpm for 10 min to separate SDS-soluble fraction (supernatant) and SDS-resistant fraction (pellet). The pellet was resuspended in 2% SDS lysis buffer (66 mM Tris·HCl, 10% glycerol, 0.005% Bromophenol Blue), boiled for 5 min, and centrifuged at 13000 rpm for 10 min.

### Immunofluorescence and TUNEL staining

The sperm were spread on glass slides for morphological observation or immuno-staining. For paraffin sections, the testes were fixed in 4% paraformaldehyde (PFA) overnight at 4°C, and treated as histological analysis experiments. For immunofluorescence, testis sections (5 μm) or sperm smears were subjected to antigen retrieval with sodium citrate buffer (10 mM sodium citrate, 0.05% Tween-20, pH 6.0), then blocked in ADB (1% Normal donkey serum, 0.3% BSA, 0.05% Triton X-100) for 60 min at room temperature, and incubated with the primary antibodies in ADB overnight at 4°C. The primary antibodies used are as follows: rabbit anti-HK1 antibody (1: 500, #2024, Cell Signaling Technology, USA) and mouse anti-α-Tubulin antibody (1: 500, 66031-1-Ig, Proteintech, USA). Subsequently, slides were washed three times with PBST (PBS with 0.1% Tween-20) and incubated with appropriate secondary antibodies with in ADB at 37°C for 60 min. The secondary antibodies used are as follows: Alexa Fluor 555 conjugated donkey anti-rabbit IgG (1: 500, A-31572, Invitrogen, USA) and Alexa Fluor 647 conjugated donkey Anti-mouse IgG (1: 500, A-31571, Invitrogen, USA). Then, slides were washed another three times followed by staining with peptide nucleic acid (1: 1000, PNA, L738, Sigma-Aldrich, USA) or MitoTracker Deep Red FM (1: 1000, M22426, Thermo Fisher Scientific, USA) before being counterstained with 1 μg/ml 4’, 6-Diamidino-2-phenylindole (1: 1000, DAPI, D1306, Invitrogen, USA).

For TUNEL analysis, assays were carried out using the In Situ Cell Death Detection Kit (#11684795910, Roche, Switzerland) following the manufacturer’s instructions. Images were acquired using confocal microscope Zeiss LSM800 or Nikon SIM A1 and microscope Olympus VS120.

Transmission electron microscopy (TEM)

TEM was performed at the Centre for Electron Microscopy of the National Institute of Biological Sciences, Beijing, following standard protocols. Briefly, the sperm were fixed with 2.5% glutaraldehyde (G5882, Sigma-Aldrich, USA) at 4°C for overnight, followed by secondary fixing with 1% OsO_4_ for 60 min on ice. Samples were then dehydrated through a series of ascending acetone solutions by the progressive lowering temperature method. The samples were then infiltrated and embedded in SPI-Pon 812 resin (Electron Microscopy Sciences). 90 nm ultrathin sections were prepared using an ultramicrotome (Leica EM UC7, Leica Microsystems, Germany), and stained with 3% uranyl acetate in 70% methanol/H_2_O for 7 min, followed by Sato’s lead for 2 min. Images were obtained on a TECNAI spirit G2 (FEI) transmission electron microscope at 120 kV.

### Immuno-electron microscopy (IEM)

Sperm were centrifuged and frozen by high-pressure freezing using a COMPACT 01 apparatus (M. Wohlwend GmbH, Switzerland) and then dehydrated by a freeze-substitution fixation process (AFS2, Leica, Germany) with 0.1% uranyl acetate in acetone. Subsequently, sperm were embedded in LR white resin, and 90 nm sections on nickel mesh were used for immunolabeling. The sections were washed in PBS for 5 min and then blocked in 2% BSA for 10 min at room temperature, and incubated with rabbit anti-HK1 (1: 20, #2024, Cell Signaling Technology, USA) in 2% BSA at 4°C overnight. Samples were washed three times with PBST and incubated with goat anti-rabbit IgG conjugated to 10 nm gold particles (1: 100, G7402, Sigma-Aldrich, USA) for 60 min at room temperature. After PBS wash, the sections were post-stained with 3% uranyl acetate in 70% methanol/H_2_O for 7 min, followed by Sato’s lead for 2 min. Images were obtained on a TECNAI spirit G2 (FEI) transmission electron microscope.

### Analysis of acrosome reaction rates

Mouse cauda epididymis was pierced and incubated in 400 μl TYH medium for 10 min, 30 min, and 60 min at 37°C under 5% CO_2_. Using two ways to induce the acrosome reaction: 1) Sperm were released from the cauda epididymis in TYH medium for 10 min and then treated with a calcium ionophore A23187 (C7522, Sigma-Aldrich, USA) at a final concentration of 10 μM for 50 min. 2) Sperm were capacitated in TYH medium at 37°C under 5% CO_2_ for 30 min. Then capacitated sperm were incubated with 5 μM progesterone (P0130, Sigma-Aldrich, USA) in TYH medium at 37°C for 30 min. The sperm were fixed in 2% PFA, spotted onto glass slides and air-dried. Acrosome status was evaluated by staining with Coomassie Brilliant Blue (0.22% R-250, 50% methanol, 10% glacial acetic acid, 40% water) for 5 min. For each glass slide, at least three different fields and 300 sperm were examined.

### RNA purification and quantitative real-time PCR (qRT-PCR)

Tissues or sperm were homogenized in TRIZOL reagent (#15596026, Invitrogen, USA) and chloroform was added to extract twice. After centrifuging, the upper aqueous phase was transferred into isopropanol followed by another centrifuging to obtain RNA pellet. Pellet was washed two times using 75% ethanol, air-dried, and solubilized in nuclease-free water. Reverse transcription was performed using PrimeScript RT reagent kit with gDNA Eraser (RR047A, TaKaRa, China) according to the manufacturer’s instructions. qRT-PCR was performed using SYBR Green master mix (DRR420A, TaKaRa, China) using Bio-Rad CFX96 Real-Time System. Relative mRNA expression levels were calculated using the comparative CT method (normalized to the *Gapdh*). All of the experiments were repeated independently at least three times. The primers used are listed in the S1 Table.

### LC-MS-based targeted metabolomics analysis

Sperm were isolated from cauda epididymis and incubated for 2 h under capacitated condition in 500 μl TYH medium at 37°C in 5% CO_2_. After centrifugation at 13000 rpm, 4°C for 5 min, the pellet was washed three times with PBS, added 1 ml precooled lysis buffer (methanol: acetonitrile: water = 40: 40: 20[vol/vol]), disintegrated by physical shock for 3 min and incubated at -80°C for 60 min. After centrifugation at 13000 rpm, 4°C for 20 min, the supernatant was concentrated in a vacuum evaporation. Sample dry powder was stocked at -80°C until further analysis.

The LC-MS analysis was performed by a Vanquish UHPLC coupled to a Q Exactive HFX mass spectrometer (Thermo Fisher Scientific, Bremen, Germany). An Acquity BEH Amide column (2.1x100 mm, 1.7 μm) (Waters, Milford, MA) was used for UHPLC separation. The mobile phases consisted of 25 mM ammonium acetate and 25 mM ammonium hydroxide (pH 9.75) in water (A) and acetonitrile (B). The following gradient was applied: 0–0.5 min, 95% B; 0.5–7 min, 95–65% B; 7–8 min, 65–40% B; 8–9 min, 40% B; 9–9.1, 40–95% B; 9.1–12 min, 95% B. The flow rate was 0.5 ml/min, and the column temperature was 40°C. Full-scan mass spectra were acquired in the range of *m/z* 66.7 to 1000 with the following ESI source settings: spray voltage: 3.5 kV (positive mode) or 2.5 kV (negative mode), auxiliary gas heater temperature: 380°C, capillary temperature: 320°C, sheath gas flow rate: 30 units, auxiliary gas flow: 10 units. MS1 scan parameters included resolution 60000, AGC target 3e6, and maximum injection time of 200 ms. Data processing was performed with Thermo Xcalibur software (4.2).

### Sperm ATP level measurements

Sperm ATP levels were measured using CellTiter-Glo Luminescent Cell Viability Assay (G7570, Promega, USA) [49]. Sperm were incubated in TYH medium at 37°C in 5% CO_2_ in humidified air and assayed after 10 min and 2 h, and adjusted to about 5x10^6^ sperm/ml. 50 μl of each sample were transferred to wells in a white 96-well microtiter plate. Subsequently, 50 μl CellTiter-Glo Reagent was added, and the mixture was shaken in the dark for 10 min at room temperature (24–26°C), Luminescence was recorded with a Tecan GENios Pro plate reader.

### Analysis of sperm migration into the oviduct

Superovulated wild-type female mice were caged with *Hk1s*^+*/*−^ and *Hk1s*^−*/*−^ males and checked for the formation of vaginal plugs every 30 min. Female mice were killed 2 hours after vaginal plug formation. Bilateral oviducts were removed and flushed out the sperm with TYH using a 26-gauge needle. The number of sperm was counted using a hematocytometer. For H&E analysis, uterus and oviduct junction of plugged females was excised and fixed 2 h after coitus. Serial sections containing UTJ were made from paraffin-embedded tissue, stained with H&E, and examined for the presence of sperm under a bright-field microscope system (Leica Microsystems, Germany).

### *In vitro* fertilization (IVF)

Sperm collected from the cauda epididymis were incubated in a drop of TYH medium for 60 min at 37°C under 5% CO_2._ Oocytes were collected from the superovulated females, treated with hyaluronidase (H3757, Sigma-Aldrich, USA) for 10 min to remove the cumulus cells (cumulus-free oocytes) or with Tyrode’s salt solution (T1788, Sigma-Aldrich, USA) for 1 min to remove the ZP. Sperm were added to the TYH drops that contain cumulus-intact, cumulus-free, or ZP-free oocytes at a final density of 1x10^6^ sperm/ml and incubated at 37°C under 5% CO_2._ Two-cells were counted the next day using Hoffman Modulation Contrast microscope (OLYMPUS IX71). And fertilization rate was calculated as the ratio between the number of pronuclear-stage embryos and the total number of pronuclear-stage embryos plus oocytes recovered [50]. For sperm penetration and binding assays, sperm were added to the TYH drops that contain cumulus-intact or ZP-free oocytes at a final density of 1x10^5^ sperm/ml and incubated at 37°C under 5% CO_2._ After 60 min of coincubation, oocytes were washed gently with TYH medium to remove free sperm and the sperm binding loosely.

### Statistical analysis

All data are presented as the means ± standard error mean (SEM). Significance difference was tested by using the two-tailed Student’s *t*-test (*, *P* < 0.05; **, *P* < 0.01; ***, *P* < 0.001; ns: non-significant, *P* > 0.05) using GraphPad Prism 6 (GraphPad Software). At least three mice were used in each experimental group.

## Supporting information

S1 Fig HK1(S) is absent in *Hk1s*^−/−^ testis and sperm. Western blot analysis of HK1(S) protein levels in *Hk1s*^+/−^ and *Hk1s*^−*/*−^ testis and sperm. α-Tubulin was used as a loading control.
(TIF)

S2 Fig HK1S is localized in the flagella region of step 15–16 spermatids in mouse testis. Immunostaining analysis of HK1S in *Hk1s*^+/−^ and *Hk1s*^−*/*−^ testis sections. The white boxes showed flagella of elongated spermatids in seminiferous tubules at stage VII-VIII. HK1S (red); PNA (green), as the marker of sperm acrosome; α-Tubulin (white), as the marker of sperm flagella; DAPI nuclear counterstaining of DNA (blue). Scale bar: 100 μm.
(TIF)

S3 Fig HK1(S) are not expressed in the round spermatids. (A) Images of isolated mouse round spermatids and elongating/elongated spermatids from adult testicle samples using STA-PUT velocity sedimentation. Each population is stained with PNA and DAPI to show differences in nuclear size and morphology. Scale bar: 10 μm. (B) Western blot analysis of HK1(S) protein levels in *Hk1s*^+/−^ and *Hk1s*^−*/*−^ round spermatids (RS), elongating/elongated spermatids (ES) and sperm.
(TIF)

S4 Fig *Hk1s* deficiency does not affect germ cell apoptosis. TUNEL staining of adult *Hk1s*^+*/*−^ or *Hk1s*^−*/*−^ testis and cauda epididymis sections. Scale bar: 100 μm.
(TIF)

S5 Fig Disruption of *Hk1s* has normal sperm ATP levels. Sperm ATP levels measured with The CellTiter-Glo Luminescent Cell Viability Assay for 10 min (non-capacitated condition, Non-cap) and 2 h (capacitated condition, Cap) in TYH medium. Mice number (n = 3) per genotype. Error bars: SEM. Statistics, Student’s *t*-Test. ns: non-significant.
(TIF)

S6 Fig *Hk1s*^−/−^ sperm could penetrate cumulus cell layers and bind the ZP. (A) Sperm-cumulus cell layer penetrating assay. Cumulus-intact oocytes were co-incubated with *Hk1s*^+*/*−^ and *Hk1s*^−*/*−^ capacitated sperm *in vitro* for 60 min. Scale bars: 20 μm. (B) Sperm-ZP binding assay. Cumulus-free oocytes were co-incubated with capacitated *Hk1s*^+*/*−^ and *Hk1s*^−*/*−^ capacitated sperm *in vitro* for 60 min. Scale bars: 20 μm.
(TIF)

S1 Movie Sperms from *Hk1s*^+/−^ mice incubated for 10 min in TYH. Original magnification ×200.
(MP4)

S2 Movie Sperms from *Hk1s*^−/−^ mice incubated for 10 min in TYH. Original magnification ×200.
(MP4)

S3 Movie Sperms from *Hk1s*^+/−^ mice incubated for 2 h in TYH. Original magnification ×200.
(MP4)

S4 Movie Sperms from *Hk1s*^−/−^ mice incubated for 2 h in TYH. Original magnification ×200.
(MP4)

S1 Data The numerical data for all the plots presented in Fig 1, Figs 3–7, and S5 Fig. (XLSX)

S1 raw images The raw images for all the blots presented in Figs 1, 2, 7 and S1, S3 Figs. (PDF)

S1 Table Genotyping and qRT-PCR primers. (XLSX)

## References

[1] Austin CR, Bishop MW. Capacitation of mammalian spermatozoa. Nature. 1958;181(4612):851. doi: 10.1038/181851a0 . 13526698

[2] Ferreira JJ, Cassina A, Irigoyen P, Ford M, Pietroroia S, Peramsetty N, et al. Increased mitochondrial activity upon CatSper channel activation is required for mouse sperm capacitation. Redox Biol. 2021;48:102176. doi: 10.1016/j.redox.2021.102176 . 34753004 PMC8585656

[3] Okabe M. The cell biology of mammalian fertilization. Development. 2013;140(22):4471–9. doi: 10.1242/dev.090613 . 24194470

[4] De Jesus A, Keyhani-Nejad F, Pusec CM, Goodman L, Geier JA, Stoolman JS, et al. Hexokinase 1 cellular localization regulates the metabolic fate of glucose. Mol Cell. 2022;82(7):1261–77 e9. doi: 10.1016/j.molcel.2022.02.028 . 35305311 PMC8995391

[5] Puleston DJ, Villa M, Pearce EL. Ancillary Activity: Beyond Core Metabolism in Immune Cells. Cell Metab. 2017;26(1):131–41. doi: 10.1016/j.cmet.2017.06.019 . 28683280 PMC5546226

[6] Wilson JE. Isozymes of mammalian hexokinase: structure, subcellular localization and metabolic function. J Exp Biol. 2003;206(Pt 12):2049–57. doi: 10.1242/jeb.00241 . 12756287

[7] Jamwal M, Aggarwal A, Palodi A, Sharma P, Bansal D, Maitra A, et al. A nonsense variant in the Hexokinase 1 gene (HK1) causing severe non-spherocytic haemolytic anaemia: genetic analysis exemplifies ambiguity due to multiple Isoforms. Br J Haematol. 2019;186(5):e142–e5. doi: 10.1111/bjh.15981 . 31119733

[8] Nakamura N, Shibata H, O’Brien DA, Mori C, Eddy EM. Spermatogenic cell-specific type 1 hexokinase is the predominant hexokinase in sperm. Mol Reprod Dev. 2008;75(4):632–40. doi: 10.1002/mrd.20791 . 17924400 PMC2412836

[9] Adams V, Griffin L, Towbin J, Gelb B, Worley K, McCabe ER. Porin interaction with hexokinase and glycerol kinase: metabolic microcompartmentation at the outer mitochondrial membrane. Biochem Med Metab Biol. 1991;45(3):271–91. doi: 10.1016/0885-4505(91)90032-g . 1710914

[10] Eddy EM, Toshimori K, O’Brien DA. Fibrous sheath of mammalian spermatozoa. Microsc Res Tech. 2003;61(1):103–15. doi: 10.1002/jemt.10320 . 12672126

[11] Mori C, Welch JE, Fulcher KD, O’Brien DA, Eddy EM. Unique hexokinase messenger ribonucleic acids lacking the porin-binding domain are developmentally expressed in mouse spermatogenic cells. Biol Reprod. 1993;49(2):191–203. doi: 10.1095/biolreprod49.2.191 . 8396993

[12] Nakamura N, Mori C, Eddy EM. Molecular complex of three testis-specific isozymes associated with the mouse sperm fibrous sheath: hexokinase 1, phosphofructokinase M, and glutathione S-transferase mu class 5. Biol Reprod. 2010;82(3):504–15. doi: 10.1095/biolreprod.109.080580 . 19889946 PMC2825169

[13] Ferramosca A, Zara V. Bioenergetics of mammalian sperm capacitation. Biomed Res Int. 2014;2014:902953. doi: 10.1155/2014/902953 . 24791005 PMC3984864

[14] Kuang W, Zhang J, Lan Z, Deepak R, Liu C, Ma Z, et al. SLC22A14 is a mitochondrial riboflavin transporter required for sperm oxidative phosphorylation and male fertility. Cell reports. 2021;35(3):109025. Epub 2021/04/22. doi: 10.1016/j.celrep.2021.109025 . 33882315 PMC8065176

[15] Nascimento JM, Shi LZ, Tam J, Chandsawangbhuwana C, Durrant B, Botvinick EL, et al. Comparison of glycolysis and oxidative phosphorylation as energy sources for mammalian sperm motility, using the combination of fluorescence imaging, laser tweezers, and real-time automated tracking and trapping. Journal of cellular physiology. 2008;217(3):745–51. doi: 10.1002/jcp.21549 . 18683212 PMC3501448

[16] Mukai C, Okuno M. Glycolysis plays a major role for adenosine triphosphate supplementation in mouse sperm flagellar movement. Biol Reprod. 2004;71(2):540–7. doi: 10.1095/biolreprod.103.026054 . 15084484

[17] Mori C, Nakamura N, Welch JE, Gotoh H, Goulding EH, Fujioka M, et al. Mouse spermatogenic cell-specific type 1 hexokinase (mHk1-s) transcripts are expressed by alternative splicing from the mHk1 gene and the HK1-S protein is localized mainly in the sperm tail. Mol Reprod Dev. 1998;49(4):374–85. doi: 10.1002/(SICI)1098-2795(199804)49:4&lt;374::AID-MRD4&gt;3.0.CO;2-K . 9508088

[18] Vemuganti SA, Bell TA, Scarlett CO, Parker CE, de Villena FP, O’Brien DA. Three male germline-specific aldolase A isozymes are generated by alternative splicing and retrotransposition. Dev Biol. 2007;309(1):18–31. doi: 10.1016/j.ydbio.2007.06.010 . 17659271

[19] Welch JE, Schatte EC, O’Brien DA, Eddy EM. Expression of a glyceraldehyde 3-phosphate dehydrogenase gene specific to mouse spermatogenic cells. Biol Reprod. 1992;46(5):869–78. doi: 10.1095/biolreprod46.5.869 . 1375514

[20] Bunch DO, Welch JE, Magyar PL, Eddy EM, O’Brien DA. Glyceraldehyde 3-phosphate dehydrogenase-S protein distribution during mouse spermatogenesis. Biol Reprod. 1998;58(3):834–41. doi: 10.1095/biolreprod58.3.834 . 9510974

[21] Millan JL, Driscoll CE, LeVan KM, Goldberg E. Epitopes of human testis-specific lactate dehydrogenase deduced from a cDNA sequence. Proc Natl Acad Sci U S A. 1987;84(15):5311–5. doi: 10.1073/pnas.84.15.5311 . 2440048 PMC298845

[22] Boer PH, Adra CN, Lau YF, McBurney MW. The testis-specific phosphoglycerate kinase gene pgk-2 is a recruited retroposon. Mol Cell Biol. 1987;7(9):3107–12. doi: 10.1128/mcb.7.9.3107-3112.1987 . 2823118 PMC367943

[23] Krisfalusi M, Miki K, Magyar PL, O’Brien DA. Multiple glycolytic enzymes are tightly bound to the fibrous sheath of mouse spermatozoa. Biol Reprod. 2006;75(2):270–8. doi: 10.1095/biolreprod.105.049684 . 16687649

[24] Williams AC, Ford WC. The role of glucose in supporting motility and capacitation in human spermatozoa. J Androl. 2001;22(4):680–95. . 11451366

[25] Narisawa S, Hecht NB, Goldberg E, Boatright KM, Reed JC, Millan JL. Testis-specific cytochrome c-null mice produce functional sperm but undergo early testicular atrophy. Mol Cell Biol. 2002;22(15):5554–62. doi: 10.1128/MCB.22.15.5554-5562.2002 . 12101247 PMC133957

[26] Travis AJ, Jorgez CJ, Merdiushev T, Jones BH, Dess DM, Diaz-Cueto L, et al. Functional relationships between capacitation-dependent cell signaling and compartmentalized metabolic pathways in murine spermatozoa. J Biol Chem. 2001;276(10):7630–6. doi: 10.1074/jbc.M006217200 . 11115497

[27] Urner F, Leppens-Luisier G, Sakkas D. Protein tyrosine phosphorylation in sperm during gamete interaction in the mouse: the influence of glucose. Biol Reprod. 2001;64(5):1350–7. doi: 10.1095/biolreprod64.5.1350 . 11319138

[28] Fraser LR, Quinn PJ. A glycolytic product is obligatory for initiation of the sperm acrosome reaction and whiplash motility required for fertilization in the mouse. J Reprod Fertil. 1981;61(1):25–35. doi: 10.1530/jrf.0.0610025 . 7452624

[29] Hoshi K, Tsukikawa S, Sato A. Importance of Ca2+, K+ and glucose in the medium for sperm penetration through the human zona pellucida. Tohoku J Exp Med. 1991;165(2):99–104. doi: 10.1620/tjem.165.99 . 1812597

[30] Miki K, Qu W, Goulding EH, Willis WD, Bunch DO, Strader LF, et al. Glyceraldehyde 3-phosphate dehydrogenase-S, a sperm-specific glycolytic enzyme, is required for sperm motility and male fertility. Proc Natl Acad Sci U S A. 2004;101(47):16501–6. doi: 10.1073/pnas.0407708101 . 15546993 PMC534542

[31] Odet F, Duan C, Willis WD, Goulding EH, Kung A, Eddy EM, et al. Expression of the gene for mouse lactate dehydrogenase C (Ldhc) is required for male fertility. Biol Reprod. 2008;79(1):26–34. doi: 10.1095/biolreprod.108.068353 . 18367675 PMC2574787

[32] Danshina PV, Geyer CB, Dai Q, Goulding EH, Willis WD, Kitto GB, et al. Phosphoglycerate kinase 2 (PGK2) is essential for sperm function and male fertility in mice. Biol Reprod. 2010;82(1):136–45. doi: 10.1095/biolreprod.109.079699 . 19759366 PMC2802118

[33] Cao W, Gerton GL, Moss SB. Proteomic profiling of accessory structures from the mouse sperm flagellum. Mol Cell Proteomics. 2006;5(5):801–10. doi: 10.1074/mcp.M500322-MCP200 . 16452089

[34] Miyata H, Shimada K, Morohoshi A, Oura S, Matsumura T, Xu Z, et al. Testis-enriched kinesin KIF9 is important for progressive motility in mouse spermatozoa. FASEB J. 2020;34(4):5389–400. doi: 10.1096/fj.201902755R . 32072696 PMC7136151

[35] Morohoshi A, Miyata H, Tokuhiro K, Iida-Norita R, Noda T, Fujihara Y, et al. Testis-enriched ferlin, FER1L5, is required for Ca(2+)-activated acrosome reaction and male fertility. Sci Adv. 2023;9(4):eade7607. doi: 10.1126/sciadv.ade7607 . 36696506 PMC9876558

[36] Stival C, Puga Molina Ldel C, Paudel B, Buffone MG, Visconti PE, Krapf D. Sperm Capacitation and Acrosome Reaction in Mammalian Sperm. Adv Anat Embryol Cell Biol. 2016;220:93–106. doi: 10.1007/978-3-319-30567-7_5 . 27194351

[37] Sepideh J, Reza SM, Mahdi AM, Azadeh EH, Naser A, Niknam L, et al. Tyrosine phosphorylation pattern in sperm proteins isolated from normospermic and teratospermic men. J Reprod Infertil. 2009;10(3):185–91. . 23926467 PMC3719330

[38] Kalab P, Visconti P, Leclerc P, Kopf GS. p95, the major phosphotyrosine-containing protein in mouse spermatozoa, is a hexokinase with unique properties. J Biol Chem. 1994;269(5):3810–7. . 7508920

[39] Visconti PE, Olds-Clarke P, Moss SB, Kalab P, Travis AJ, de las Heras M, et al. Properties and localization of a tyrosine phosphorylated form of hexokinase in mouse sperm. Mol Reprod Dev. 1996;43(1):82–93. doi: 10.1002/(SICI)1098-2795(199601)43:1&lt;82::AID-MRD11&gt;3.0.CO;2-6 . 8720117

[40] Krapf D, Arcelay E, Wertheimer EV, Sanjay A, Pilder SH, Salicioni AM, et al. Inhibition of Ser/Thr phosphatases induces capacitation-associated signaling in the presence of Src kinase inhibitors. J Biol Chem. 2010;285(11):7977–85. doi: 10.1074/jbc.M109.085845 . 20068039 PMC2832948

[41] Huang Z, Danshina PV, Mohr K, Qu W, Goodson SG, O’Connell TM, et al. Sperm function, protein phosphorylation, and metabolism differ in mice lacking successive sperm-specific glycolytic enzymes. Biol Reprod. 2017;97(4):586–97. doi: 10.1093/biolre/iox103 . 29025010

[42] Nakamura N, Dai Q, Williams J, Goulding EH, Willis WD, Brown PR, et al. Disruption of a spermatogenic cell-specific mouse enolase 4 (eno4) gene causes sperm structural defects and male infertility. Biol Reprod. 2013;88(4):90. doi: 10.1095/biolreprod.112.107128 . 23446454 PMC4013874

[43] Amaral A, Castillo J, Estanyol JM, Ballesca JL, Ramalho-Santos J, Oliva R. Human sperm tail proteome suggests new endogenous metabolic pathways. Molecular & cellular proteomics: MCP. 2013;12(2):330–42. doi: 10.1074/mcp.M112.020552 . 23161514 PMC3567857

[44] Swegen A, Curry BJ, Gibb Z, Lambourne SR, Smith ND, Aitken RJ. Investigation of the stallion sperm proteome by mass spectrometry. Reproduction (Cambridge, England). 2015;149(3):235–44. Epub 2014/12/17. doi: 10.1530/REP-14-0500 . 25504869

[45] Stival C, Ritagliati C, Xu X, Gervasi MG, Luque GM, Baro Graf C, et al. Disruption of protein kinase A localization induces acrosomal exocytosis in capacitated mouse sperm. J Biol Chem. 2018;293(24):9435–47. doi: 10.1074/jbc.RA118.002286 . 29700114 PMC6005427

[46] Mori C, Nakamura N, Welch JE, Shiota K, Eddy EM. Testis-specific expression of mRNAs for a unique human type 1 hexokinase lacking the porin-binding domain. Mol Reprod Dev. 1996;44(1):14–22. doi: 10.1002/(SICI)1098-2795(199605)44:1&lt;14::AID-MRD2&gt;3.0.CO;2-W . 8722688

[47] Li YS, Meng RR, Chen X, Shang CL, Li HB, Zhang TJ, et al. Generation of H11-albumin-rtTA Transgenic Mice: A Tool for Inducible Gene Expression in the Liver. G3 (Bethesda). 2019;9(2):591–9. doi: 10.1534/g3.118.200963 . 30591434 PMC6385985

[48] Da Ros M, Lehtiniemi T, Olotu O, Meikar O, Kotaja N. Enrichment of Pachytene Spermatocytes and Spermatids from Mouse Testes Using Standard Laboratory Equipment. Journal of visualized experiments: JoVE. 2019;(151). Epub 2019/10/15. doi: 10.3791/60271 . 31609338

[49] Chen Y, Chen X, Zhang H, Sha Y, Meng R, Shao T, et al. TBC1D21 is an essential factor for sperm mitochondrial sheath assembly and male fertilitydouble dagger. Biol Reprod. 2022;107(2):619–34. doi: 10.1093/biolre/ioac069 . 35403672

[50] Zhang H, Li Y, Cui K, Chen X, Shang C, Min W, et al. Male fertility in Mus musculus requires the activity of TRYX5 in sperm migration into the oviduct. J Cell Physiol. 2020;235(9):6058–72. doi: 10.1002/jcp.29534 . 32020604

