# Supplementary figures and images for: Spermatogenic cell-specific type 1 hexokinase (HK1S) is essential for capacitation-associated increase in tyrosine phosphorylation and male fertility in mice
Source: PLoS Genet. 2024 Jul 29;20(7):e1011357. doi: 10.1371/journal.pgen.1011357 (PMC11285943; doi:10.1371/journal.pgen.1011357)

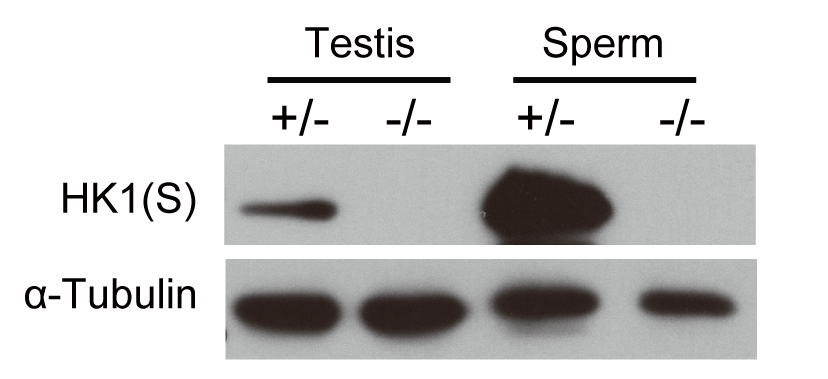

Supplement: S1 Fig — Western blot analysis of HK1(S) protein levels in Hk1s+/− and Hk1s−/− testis and sperm. α-Tubulin was used as a loading control. (TIF) [file pgen.1011357.s001.tif]

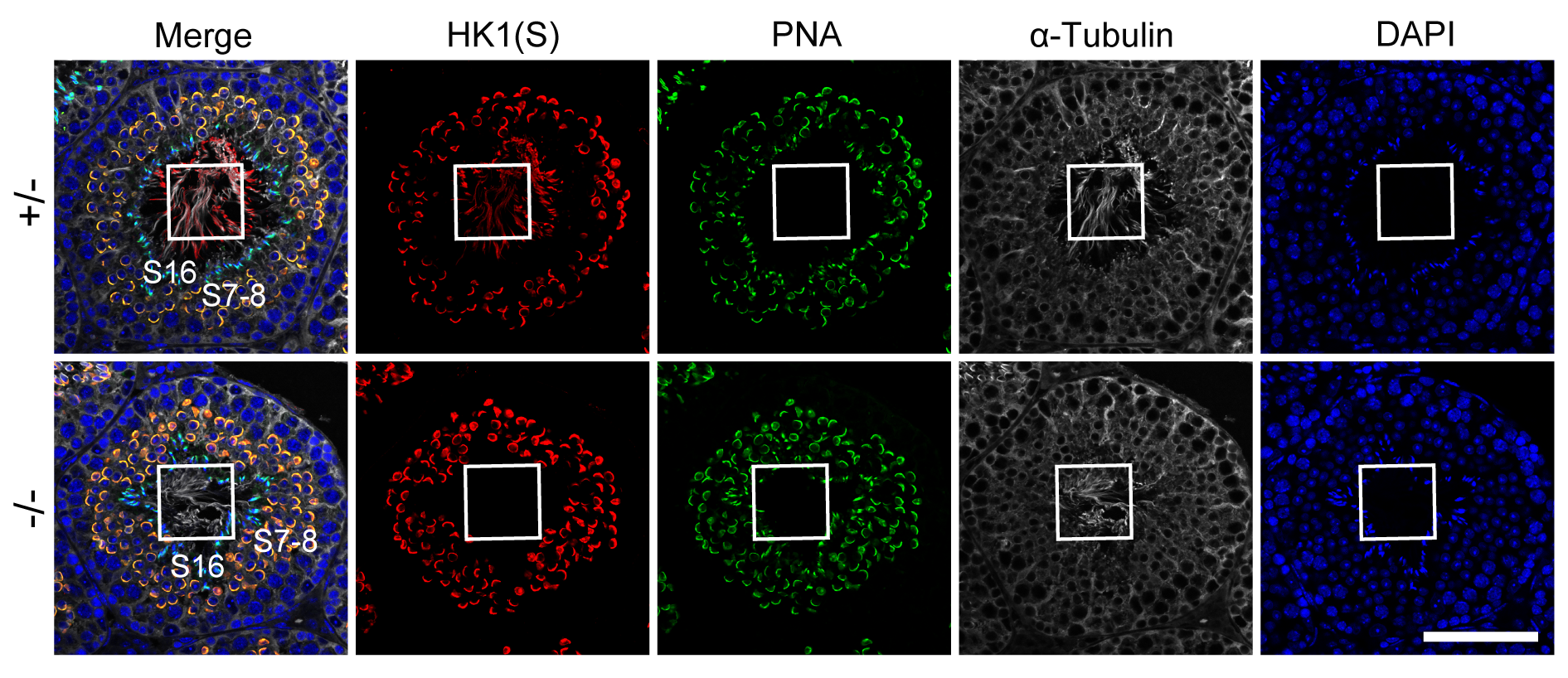

Supplement: S2 Fig — Immunostaining analysis of HK1S in Hk1s+/− and Hk1s−/− testis sections. The white boxes showed flagella of elongated spermatids in seminiferous tubules at stage VII-VIII. HK1S (red); PNA (green), as the marker of sperm acrosome; α-Tubulin (white), as the marker of sperm flagella; DAPI nuclear counterstaining of DNA (blue). Scale bar: 100 μm. (TIF) [file pgen.1011357.s002.tif]

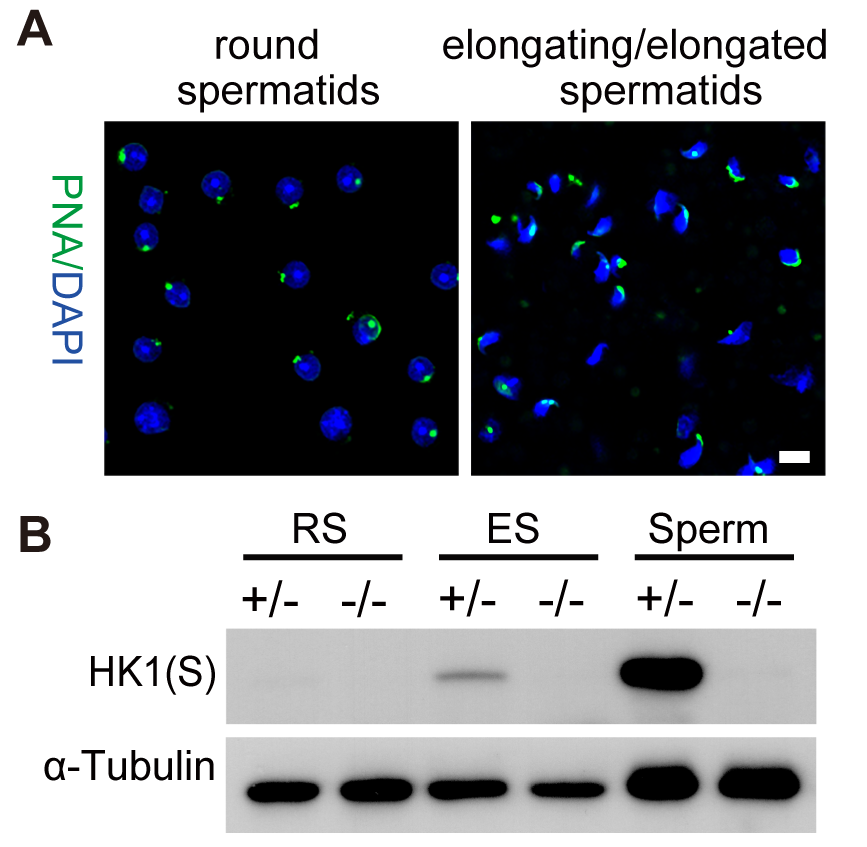

Supplement: S3 Fig — (A) Images of isolated mouse round spermatids and elongating/elongated spermatids from adult testicle samples using STA-PUT velocity sedimentation. Each population is stained with PNA and DAPI to show differences in nuclear size and morphology. Scale bar: 10 μm. (B) Western blot analysis of HK1(S) protein levels in Hk1s+/− and Hk1s−/− round spermatids (RS), elongating/elongated spermatids (ES) and sperm. (TIF) [file pgen.1011357.s003.tif]

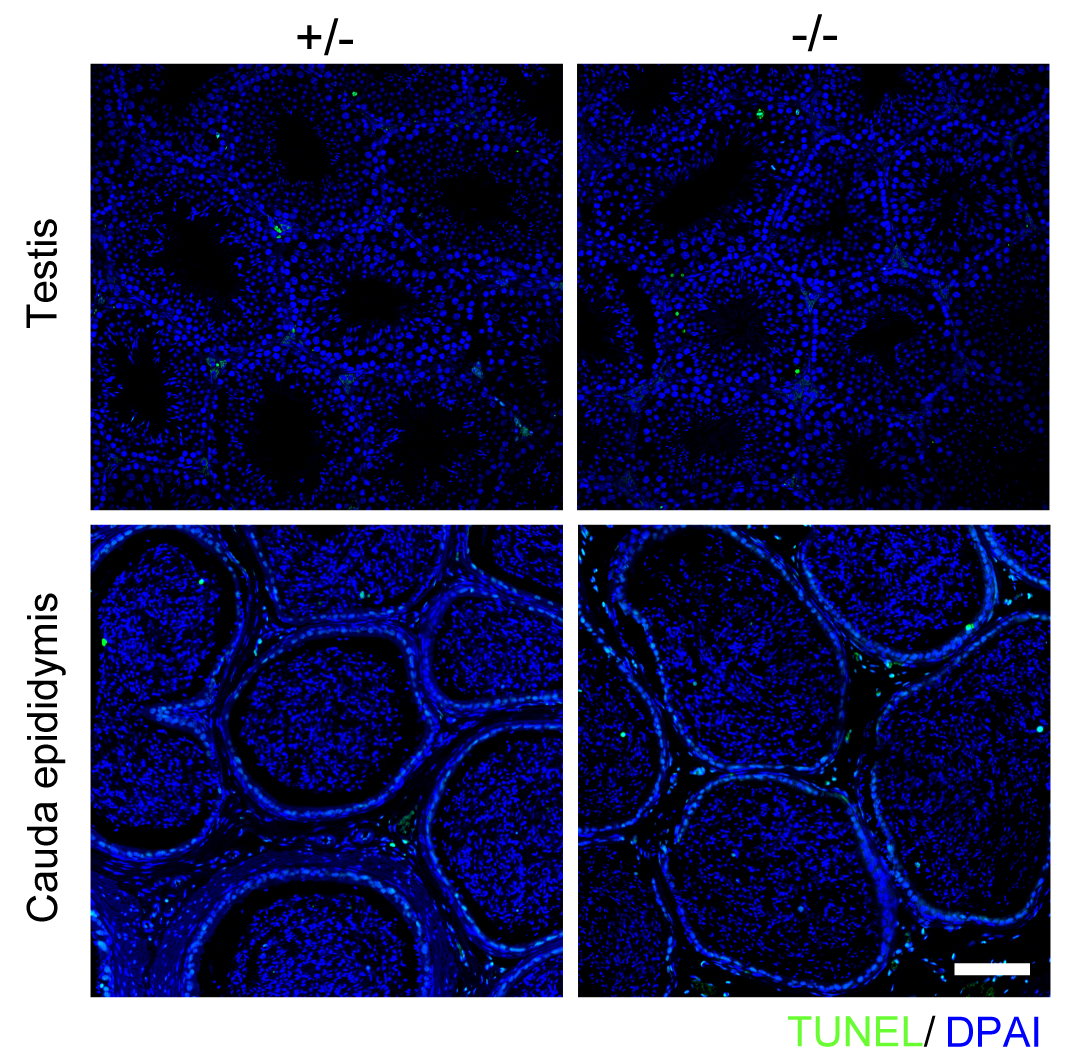

Supplement: S4 Fig — TUNEL staining of adult Hk1s+/− or Hk1s−/− testis and cauda epididymis sections. Scale bar: 100 μm. (TIF) [file pgen.1011357.s004.tif]

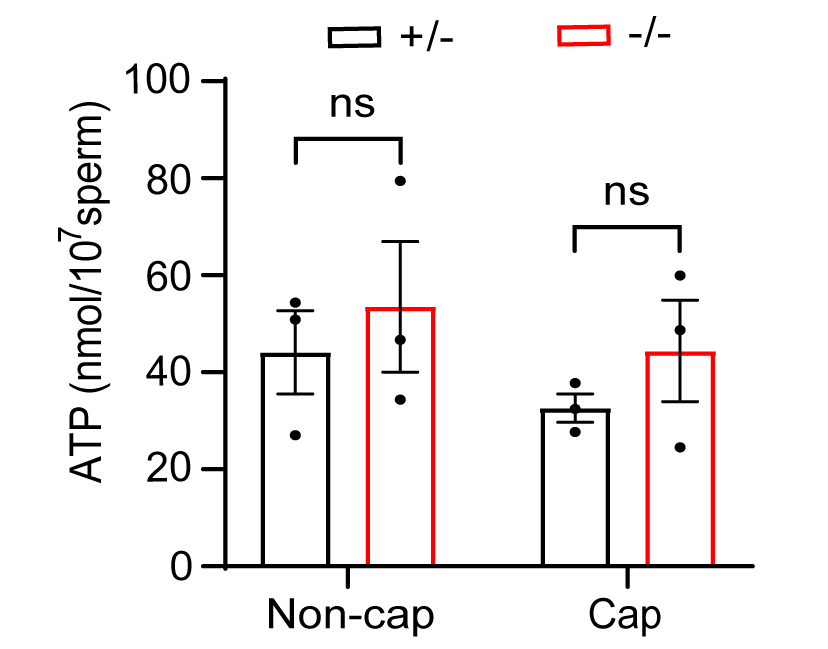

Supplement: S5 Fig — Sperm ATP levels measured with The CellTiter-Glo Luminescent Cell Viability Assay for 10 min (non-capacitated condition, Non-cap) and 2 h (capacitated condition, Cap) in TYH medium. Mice number (n = 3) per genotype. Error bars: SEM. Statistics, Student’s t-Test. ns: non-significant. (TIF) [file pgen.1011357.s005.tif]

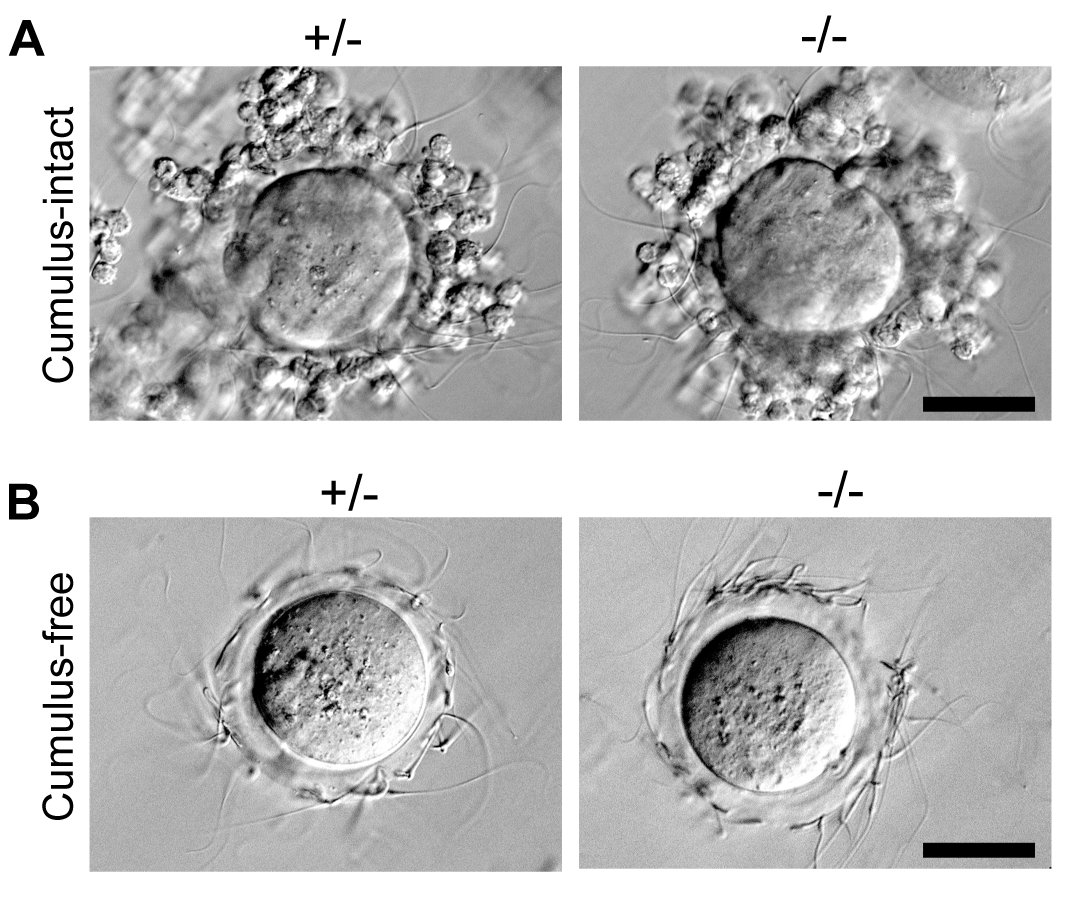

Supplement: S6 Fig — (A) Sperm-cumulus cell layer penetrating assay. Cumulus-intact oocytes were co-incubated with Hk1s+/− and Hk1s−/− capacitated sperm in vitro for 60 min. Scale bars: 20 μm. (B) Sperm-ZP binding assay. Cumulus-free oocytes were co-incubated with capacitated Hk1s+/− and Hk1s−/− capacitated sperm in vitro for 60 min. Scale bars: 20 μm. (TIF) [file pgen.1011357.s006.tif]
