# Supplementary material for: Spermatogenic cell-specific type 1 hexokinase (HK1S) is essential for capacitation-associated increase in tyrosine phosphorylation and male fertility in mice
Source: PLoS Genet. 2024 Jul 29;20(7):e1011357. doi: 10.1371/journal.pgen.1011357 (PMC11285943; doi:10.1371/journal.pgen.1011357)
Supplement: S1 raw images — (PDF) [file pgen.1011357.s012.pdf]

S1_raw_images

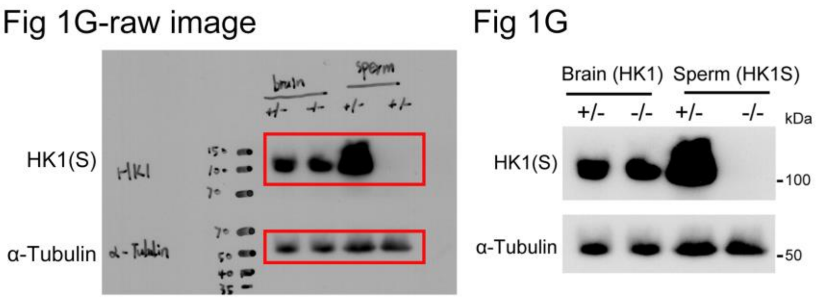

Fig 1G raw images of immunoblotting. PVDF membrane was cut into two pieces to incubate with anti-HK1(S) and anti-acetylated Tubulin for immunoblotting. Red boxes indicate images shown in relevant figures.

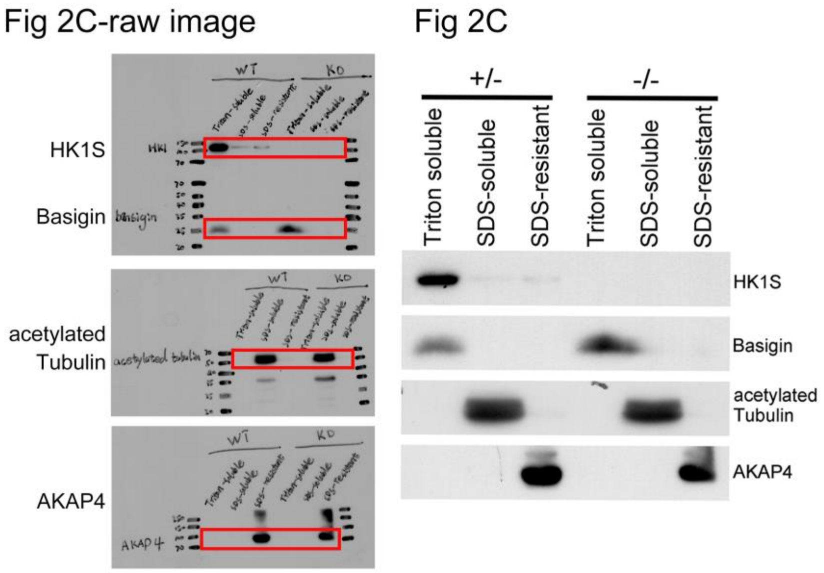

Fig 2C raw images of immunoblotting. PVDF membranes were cut into several small pieces to incubate with anti-HK1(S), anti-Basigin, anti-acetylated Tubulin, and anti-AKAP4 for immunoblotting. Red boxes indicate images shown in relevant figures.

Fig 7A-raw image

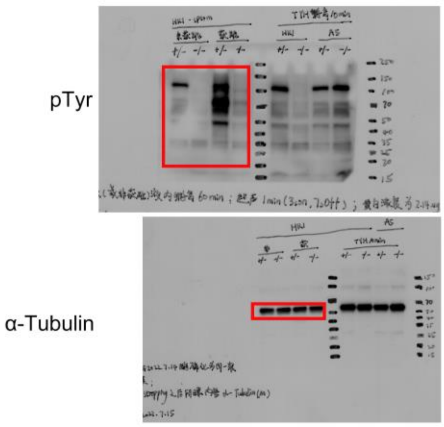

Fig 7A

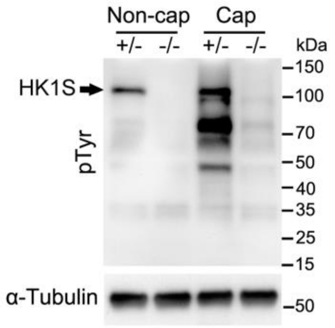

Fig 7C-raw image

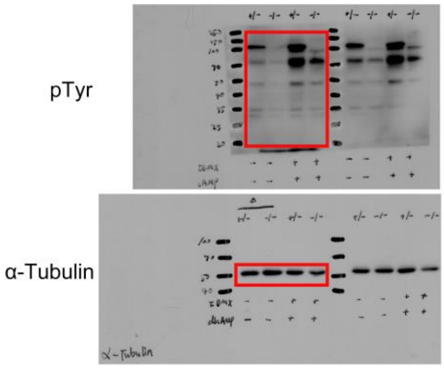

Fig 7C

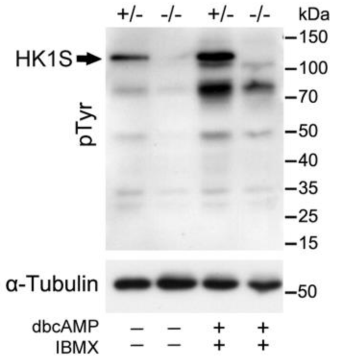

Fig 7A and 7C raw images of immunoblotting. PVDF membranes were incubated with anti-phosphotyrosine clone 4G10 for immunoblotting. The membrane was washed with stripping buffer and incubated with anti-acetylated Tubulin. Red boxes indicate images shown in relevant figures.

S1 Fig-raw image

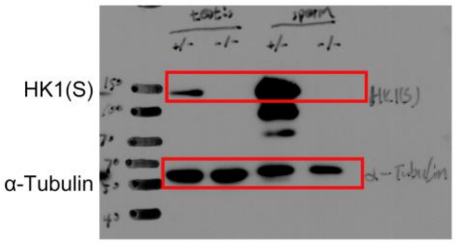

S1 Fig

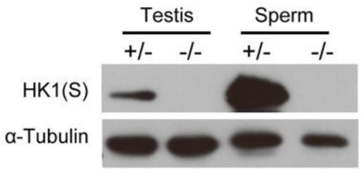

S1 Fig raw images of immunoblotting. PVDF membrane was cut into two

pieces to incubate with anti-HK1 and anti-acetylated Tubulin for immunoblotting.

Red boxes indicate images shown in relevant figures.

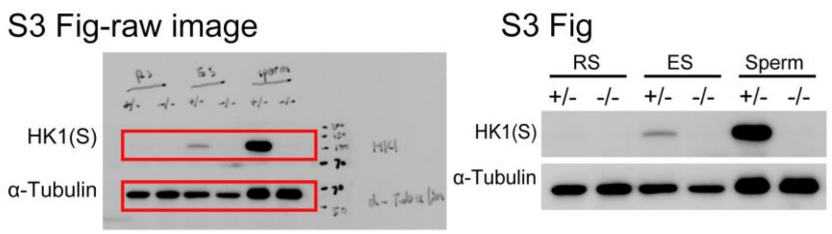

S3 Fig raw images of immunoblotting. PVDF membrane was cut into two pieces to incubate with anti-HK1 and anti-acetylated Tubulin for immunoblotting. Red boxes indicate images shown in relevant figures.
